# Regression of Breast Cancer Metastases Following Treatment with Irradiated SV-BR-1-GM, a GM-CSF Overexpressing Breast Cancer Cell Line: Intellectual Property and Immune Markers of Response

**DOI:** 10.2174/1574892817666220518123331

**Published:** 2023-12-28

**Authors:** Charles L. Wiseman, Alexander Kharazi, Vivekananda G. Sunkari, Jacqueline L. Galeas, Vito Dozio, Hind Hashwah, Eva Macúchová, William V. Williams, Markus D. Lacher

**Affiliations:** 1 BriaCell Therapeutics Corporation, 2929 Arch Street, 3^rd^ Floor, Philadelphia, PA, 19104, USA;; 2 Operations Department, Biognosys AG, Wagistrasse 21, 8952, Schlieren, Switzerland;; 3 Sales and Marketing Nebion AG, Hohlstrasse 515, 8048, Zurich, Switzerland;; 4 Immunotherapy Laboratory, St. Vincent Medical Center, Los Angeles, CA, USA

**Keywords:** SV-BR-1-GM, whole-cell immunotherapy, therapeutic cancer vaccine, HLA, breast cancer, WO2017147600A1

## Abstract

**Background:**

SV-BR-1-GM, derived from a patient with grade 2 (moderately differentiated) breast cancer, is a GM-CSF-secreting breast cancer cell line with properties of antigen-presenting cells. SV-BR-1-GM and next-generation versions are covered by several pending and granted patents.

**Methods:**

We report findings from an open-label phase I, single-arm pilot study with irradiated SV-BR-1-GM cells in 3 breast and 1 ovarian cancer subjects. Inoculations were preceded by low-dose intravenous cyclophosphamide and followed by interferon-alpha2b injections into the SV-BR-1-GM inoculation sites. We assessed both cellular and humoral immune responses, and measured expression levels of SV-BR-1-GM HLA alleles.

**Results:**

Treatment was generally safe and well tolerated. Immune responses were elicited universally. Overall survival was more than 33 months for three of the four patients. As previously reported, one patient had prompt regression of metastases in lung, breast, and soft tissue. Following cessation of treatment, the patient relapsed widely, including in the brain. Upon retreatment, rapid tumor response was again seen, including complete regression of brain metastases. Consistent with a role of Class II HLA in contributing to SV-BR-1-GM’s mechanism of action, this patient allele-matched SV-BR-1-GM at the *HLA-DRB1* and *HLA-DRB3* loci. We are in the process of developing next-generation SV-BR-1-GM, expressing patient-specific HLAs. Patent applications were filed in various jurisdictions. Thus far, one is granted, in Japan.

**Conclusion:**

A whole-cell immunotherapy regimen with SV-BR-1-GM cells induced regression of metastatic breast cancer. We develop intellectual property based on SV-BR-1-GM’s predicted mechanism of action to develop additional whole-cell immunotherapies for cancer patients.

**Clinical Trial Registration:**

This clinical trial was registered under ClinicalTrials.gov Identifier NCT00095862.

## 1 INTRODUCTION

While many investigators have hoped that the immunization with tumor cells, or individual tumor-associated antigens (TAAs), might somehow be therapeutic for cancer patients, despite many clinical trials, only one therapy using this approach (PROVENGE^®^) has achieved FDA approval thus far [1-8]. Based on previous data [9-12], it could be argued that inoculating persons with advanced disease with tumor cell antigens will produce an ineffective response. However, many studies utilizing whole tumor cells as immunogens describe striking tumor regressions in occasional remarkable responders [13-20], suggesting that such vaccines can be effective in advanced cancer. Understanding the nature of the immune responses occurring in these responders should lead to the development of necessary and sufficient interventions needed to elicit such immune responses. This in turn should give rise to novel intellectual property to protect the development of these therapeutic approaches.

Here, we describe molecular and clinical findings related to the breast cancer cell lines SV-BR-1 (Saint Vincent’s Breast Cancer cell line 1) and SV-BR-1-GM (SV-BR-1 stably transfected with *CSF2*, encoding granulocyte-macrophage colony-stimulating factor (GM-CSF)). We show data on a small phase I clinical trial with three advanced breast cancer and one ovarian cancer patients using SV-BR-1-GM as whole-cell immunotherapy. Furthermore, we present a molecular analysis of SV-BR-1-GM that adds additional detail to our previous molecular study, which suggested that SV-BR-1-GM is most effective in patients with HLA matches to SV-BR-1-GM [21]. Furthermore, we tie key findings relevant to the development of next-generation whole-cell immunotherapies to the patent literature. In particular, we will discuss differences between WO2017147600A1 [22], addressing cancer cell lines overexpressing certain HLA alleles for patient matching, and 1) EP0569678A2 [23] and US5750102A [24] (“Double transfectants of the MHC genes as cellular vaccines for immune prevention of tumor metastasis”), 2) US614990-5A (“Tumor cells with increased immunogenicity and uses therefor”) [25], 3) US20100119537A1 (“Tumor Vaccine”) [26], 4) US7807186-B2 (“Tumor cells from immune-privileged sites as base cells for cell-based cancer vaccines”) [27], 5) WO2012156969A1 (“Allogeneic tumor cell vaccination”)[28], and 6)US7674456-B2 (“Breast cancer cell lines and uses thereof”) [29].

## 2 MATERIALS AND METHODS

### 2.1 Cell Lines for *Ex vivo* Experiments

This section only pertains to the *ex vivo* experiments. The preparation of the SV-BR-1-GM cells for patient inoculation is addressed elsewhere [30]. The breast cancer cell lines, SV-BR-1 and SV-BR-1 stably transfected with *CSF2*, encoding GM-CSF (SV-BR-1-GM), have been described in detail [21-22, 29-33]. SV-BR-1 and SV-BR-1-GM cells were cultured in RPMI-1640 supplemented with 10% heat-inactivated fetal bovine serum (FBS) and GlutaMAX (Thermo Fisher Scientific), PC-3 cells (ATCC, Manassas, VA, USA) in F12K supplemented with 10% FBS, and SK-MEL-24 cells (ATCC) cells in EMEM supplemented with 15% FBS. Cell culture times varied depending on the experiment and cell line. SV-BR-1-GM cells were irradiated with 100 Gy (10K rad) using a Cesium source then pelleted and resuspended in CryoStor CS10 (BioLife Solutions Inc., Bothell, WA, USA) by KBI Biopharma, Inc. (The Woodlands, TX, USA) for cryopreservation. Typically, cells to be irradiated were grown to ~75% confluence and then harvested with viability before irradiation of >95% and following irradiation of >90%. For SV-BR-1-GM, GM-CSF production was confirmed using the R&D Systems Quantikine human GM-CSF kit, following the manufacturer’s directions (R&D Systems, Minneapolis, MN, USA). Generally, the GM-CSF production rate was in the low ng per 10^6^ cells per 24 hours range.

### 2.2 Flow Cytometry

The flow cytometry raw data were acquired on a BD LSRFortessa™ cell analyzer *via* FACSDiva software (BD Biosciences, San Jose, CA, USA), and the analysis was conducted using FlowJo software (BD, Franklin Lakes, NJ, USA) or Flowing Software version 2.5.1 (Turku Bioimaging, Finland and Turku Centre for Biotechnology, Finland; software developer: Perttu Terho). All staining was performed to evaluate the binding of antibodies to cell surface molecules.

To evaluate anti-SV-BR-1 IgG (of unknown specificity) in patient sera, SV-BR-1 cells were pre-treated with Human TruStain FcX (BioLegend, San Diego, CA, USA) to block Fc receptors, then treated with patient sera diluted 1:3 or 1:25 in staining buffer (BioLegend), washed, and labeled with 1:200 diluted Goat-Anti-human IgG Alexa Fluor 488 (Thermo Fisher Scientific, Waltham, MA, USA). Herceptin IgG1/trastuzumab (BioVision, Inc.; Milpitas, CA, USA) served as a positive control since SV-BR-1 cells express high levels of ERBB2 [33]. The percent changes in the SV-BR-1 serum IgG levels relative to baseline were determined from the median fluorescent intensity (MFI) values.

For the assessment of HLA-DR cell surface levels, SV-BR-1-GM cells, seeded at 0.5 x 10^6^ cells per well (6-well format), were treated with 0, 0.5, or 5 ng/ml (approximately 1000 IU/ml) of IFN-γ (R&D Systems, Inc.; Minneapolis, MN, USA) in RPMI-1640 supplemented with 10% FBS and GlutaMAX (Thermo Fisher Scientific) for 16 hours at 37 °C, 5% CO_2_. Cells were then pre-treated with Human TruStain FcX (BioLegend) to block Fc receptors and stained with APC-conjugated anti-HLA-DR clone L243 antibody (BioLegend) in 1% FBS in PBS. In some experiments, stained cells were fixed using 4% paraformaldehyde before the flow cytometry analysis. As references, cells were either unstained or treated with APC-conjugated isotype control antibody (IgG2a, κ; BioLegend).

### 2.3 Proteomics Screen

*Sample preparation:* Three SV-BR-1 cultures grown in parallel were detached using TrypLE Express (Thermo Fisher Scientific) and the three cell pellets were submitted to Biognosys AG (Schlieren, Switzerland) for liquid chromatography (LC)-mass spectrometry (MS) analysis. Samples were processed essentially as described [34]. In short, cell pellets were resuspended in 8 M urea, 0.1 M ammonium bicarbonate, and benzonase. Samples were then reduced with 5 mM tris(2-carboxyethyl)phosphine (TCEP) and alkylated with 25 mM iodoacetamide (1 h, 37°C), diluted to 2 M urea, and digested with trypsin (1:50 enzyme to protein ratio, 16 h, 37°C). Peptides were desalted using a C18 MicroSpin plate (The Nest Group, Southborough, MA, USA), according to the manufacturer’s instructions, dried, and resuspended in 1% acetonitrile (ACN) with 0.1% formic acid (FA). Samples were then spiked with iRT kit calibration peptides (Biognosys) following the manufacturer’s instructions, and the peptide concentration was measured using a SPECTROstar Nano spectrophotometer (BGM Labtech).

*High-pH reversed-phase fractionation:* Peptide samples were pooled and ammonium hydroxide was added to reach pH 10. The fractionation was performed using a Dionex UltiMate 3000 RS pump (Thermo Fisher Scientific) on an Acquity UPLC CSH C18 1.7 µm, 2.1 x 150 mm column (Waters Corporation, Milford, MA, USA). The gradient was 1 to 40% solvent B in 30 min, with solvent A: 20 mM ammonium formate in water, and solvent B: ACN. Fractions were taken every 30 seconds and sequentially pooled into 10 fraction pools. Fractions were dried and resuspended in 17 µL 1% ACN and 0.1% FA. Before mass spectrometric analyses, samples were spiked with iRT kit calibration peptides and peptide concentration was determined using the SPECTROstar Nano spectrophotometer.

*Proteomic LC-MS/MS analysis:* Two micrograms of peptides per sample were injected into a self-packed C18 column (Dr. Maisch ReproSil Pur, 1.9 µm particle size, 120 Å pore size, 75 µm inner diameter, 50 cm length; New Objective, Woburn, MA, USA) on a Thermo Scientific Easy nLC 1200 nano liquid chromatography system connected to a Thermo Scientific QExactive HF-X mass spectrometer equipped with a standard nanoelectrospray source. LC solvents were A: 1% ACN and 0.1% FA in water, B: 15% water, and 0.1% FA in ACN. The nonlinear LC gradient was 1-55% solvent B in 120 minutes followed by 55-90% B in 10 seconds, 90% B for 10 minutes, 90-1% B in 10 seconds and 1% B for 5 minutes. For data-dependent acquisition (DDA; shotgun LC-MS/MS), a modified TOP 15 method was used [35]. For HRM data-independent acquisition (DIA), a method with one full range survey scan and 22 DIA variable windows was used [34].

*Proteomic data analysis:* The spectral library was generated by searching all DDA and DIA raw data using SpectroMine software (Biognosys). The default settings were used, and the false discovery rate on peptide and protein levels was set to 1%. A human UniProt FASTA database (*Homo sapiens*, 2019-07-01) was used for the search engine, allowing for 2 missed cleavages, one fixed modification (carbamidomethylation of cysteine), and three variable modifications (N-term acetylation, methionine oxidation, and phosphorylation (STY)). DIA raw data were analyzed using Spectronaut Software (Biognosys) using default settings with the following modifications: data were filtered using q-value sparse and local normalization was used.

### 2.4 RNA-seq

SV-BR-1 and SV-BR-1-GM cells, seeded at 1 x 10^6^ cells per well (6-well format), were treated with 50 ng/ml (approximately 1000 IU/ml) of IFN-γ (R&D Systems, Inc.) in RPMI-1640 supplemented with 10% FBS and GlutaMAX (Thermo Fisher Scientific) for 16 hours at 37 °C, 5% CO_2_. Thereafter, RNA was extracted (RNeasy Mini kit; Qiagen, Hilden, Germany) and submitted to Novogene (Sacramento, CA, USA) for RNA-seq processing. In short, cDNA libraries were constructed with 250~300 bp inserts from mRNA isolated using poly-T oligo magnetic beads followed by random fragmentation. Samples were analyzed at 30M raw reads/sample and reported as 150 bp paired-end reads. To estimate transcript levels, kallisto [36, 37] was employed for pseudoalignment and sleuth [38] for normalization. Estimated gene-level expression levels were obtained using sleuth with protein-coding transcripts available *via* BioMart (Ensembl; www.ensembl.org) [39]. The index file for kallisto was built with the transcript sequences of the Genome Reference Consortium Human Build 38 (GRCh38), available as a FASTA file from: Ensembl.org (ftp://ftp.ensembl.org/pub/current_fasta/homo_sapiens/cdna/Homo_sapiens.GRCh38.cdna.all.fa.gz). However, to avoid interference of sequences from HLA alleles absent in SV-BR-1 and SV-BR-1-GM cells but present in the FASTA file, *HLA-A*, *HLA-B*, *HLA-C*, *HLA-DRA, HLA-DRB1, HLA-DRB3, HLA-DRB4, HLA-DRB5*, *HLA-DQA1*, *HLA-DQB1*, *HLA-DPA1*, and *HLA-DPB1*transcripts (Ensembl Transcript IDs shown in Supplementary Data Sheet **1**) were removed from the FASTA file and replaced with the coding sequences (CDS) of the corresponding alleles detected in SV-BR-1 by DNA-based next-generation sequencing (HistoGenetics LLC, Ossining, NY, USA) (HLA-A*11:01:01, HLA-A*24:02:01, HLA-B*35: 08:01, HLA-B*55:01:01, HLA-C*01:02:01, HLA-C*04:01: 01, HLA-DRA*01:01:01, HLA-DRA*01:02:02, HLA-DRB1 *11:04:01, HLA-DRB1*13:03:01, HLA-DRB3* 01:01:02, HLA-DRB3*02:02:01, HLA-DQA1*05:05:01, HLA-DQB1* 03:01:01, HLA-DPA1*01:03:01 and HLA-DPB1*04:01:01). Though not formally assessed, given that SV-BR-1-GM cells were derived from SV-BR-1, SV-BR-1-GMcells were assumed to contain the exact same HLA alleles as SV-BR-1. To assess effects of untranslated regions (UTRs) on presumptive gene expression levels, full-length transcript sequences were extracted from the genomic sequences in the IPD-IMGT/HLA Database (https://www.ebi.ac.uk/ipd/imgt/hla/) of HLA-A*11:01:01:01 (ID: HLA00043.1), HLA-A*24:02:01:01 (ID: HLA00050.1), HLA-DRB3*01:01:02:01 (ID: HLA00887.4) and HLA-DRB3*02:02:01:02 (ID: HL A06593), or obtained from the Ensembl database (ENST000-00383126.7 for HLA-DRB3*01:01:02:02 or HLA-DRB3*01: 01:02:01, and ENST00000307137.11 and ENST00000426-847.2 for HLA-DRB3*02:02:01:02), and used instead of the corresponding CDS sequences in the FASTA file. Transcripts were considered expressed when their normalized transcript per million (TPM) values were above approximately 1 [40].

### 2.5 Quantitative RT-PCR

SV-BR-1-GM, PC-3, and SK-MEL-24 (ATCC) cells were treated for 16 hours with vehicle (medium) or 50 ng/ml (approximately 1000 IU/ml) of IFN-γ (R&D Systems, Inc.), followed by RNA extraction (PureLink™ RNA Mini Kit; Thermo Fisher Scientific) and cDNA synthesis (Verso cDNA Synthesis Kit; Thermo Fisher Scientific). Real-time polymerase chain reactions (PCRs) were carried out on an ABI 7500 instrument (Applied Biosystems/Thermo Fisher Scientific) employing SYBR™ Select Master Mix (Thermo Fisher Scientific) and 200 nM of each primer. For the primer specificity validation of the HLA-DRB1*13:03:01 primer set, real-time PCR samples post-run were analyzed on an E-Gel system (E-Gel Power Snap; Thermo Fisher Scientific) using a 1.2% SYBR Safe agarose gel and an E-Gel 1 Kb Plus DNA Ladder (both Thermo Fisher Scientific). Primer sequences: 5’-TGG-GGCGGCCTGATGAGGA-3’ and 5’-TTAGG ATGGACT-CGCCGC-3’for HLA-DRB1*11:04:01, 5’-GAG CTGGGGC-GGCCTAGC-3’and 5’-TTAGGATGGACTC GCCGC-3’ for HLA-DRB1*13:03:01, 5’-GAGCTGGGGC GGCCTGTC-3’ and 5’-TGAGGATGGACTCGCCGC-3’ for HLA-DRB3* 01:01:02, 5’-TGGGGCGGCCTGA TGCCG-3’ and 5’-TGA-GGATGGACTCGCCGC-3’ for HLA-DRB3*02:02:01, and 5’-GATCAAGATCATTGC TCCTC-3’ and 5’-GGTTT-TGT CAAGAAAGGGTG-3’ for *ACTB* (reference, control).

### 2.6 Gene Signature Meta-analysis Distinguishing Grade 1 or 2 from Grade 3 Breast Cancer

We used GENEVESTIGATOR^®^ (https://genevestigator.com) [41-42] to identify breast cancer studies with grading information to identify signatures of genes expressed either ≥2 times *higher* or ≥2 times *lower* in grade 3 tumors compared to grade 1 or 2 tumors, using a false-discovery rate (FDR) of 0.01 (See Supplementary Data Sheet **2** for detailed methods).

### 2.7 Clinical Study Design

This was an open-label, single-arm trial not only designed to assess safety, but also to minimize the number of patients at risk if there was no evidence of activity.

*Study Plan and Intervention:* Written informed consent was obtained from each subject. Baseline studies included computed tomography, MRI when indicated, bone scans, complete blood count with differential (CBC), chemistry profile, urinalysis, T-B subsets, Hep B screen, CEA, and CA 27.29.

**Fig. (1):**
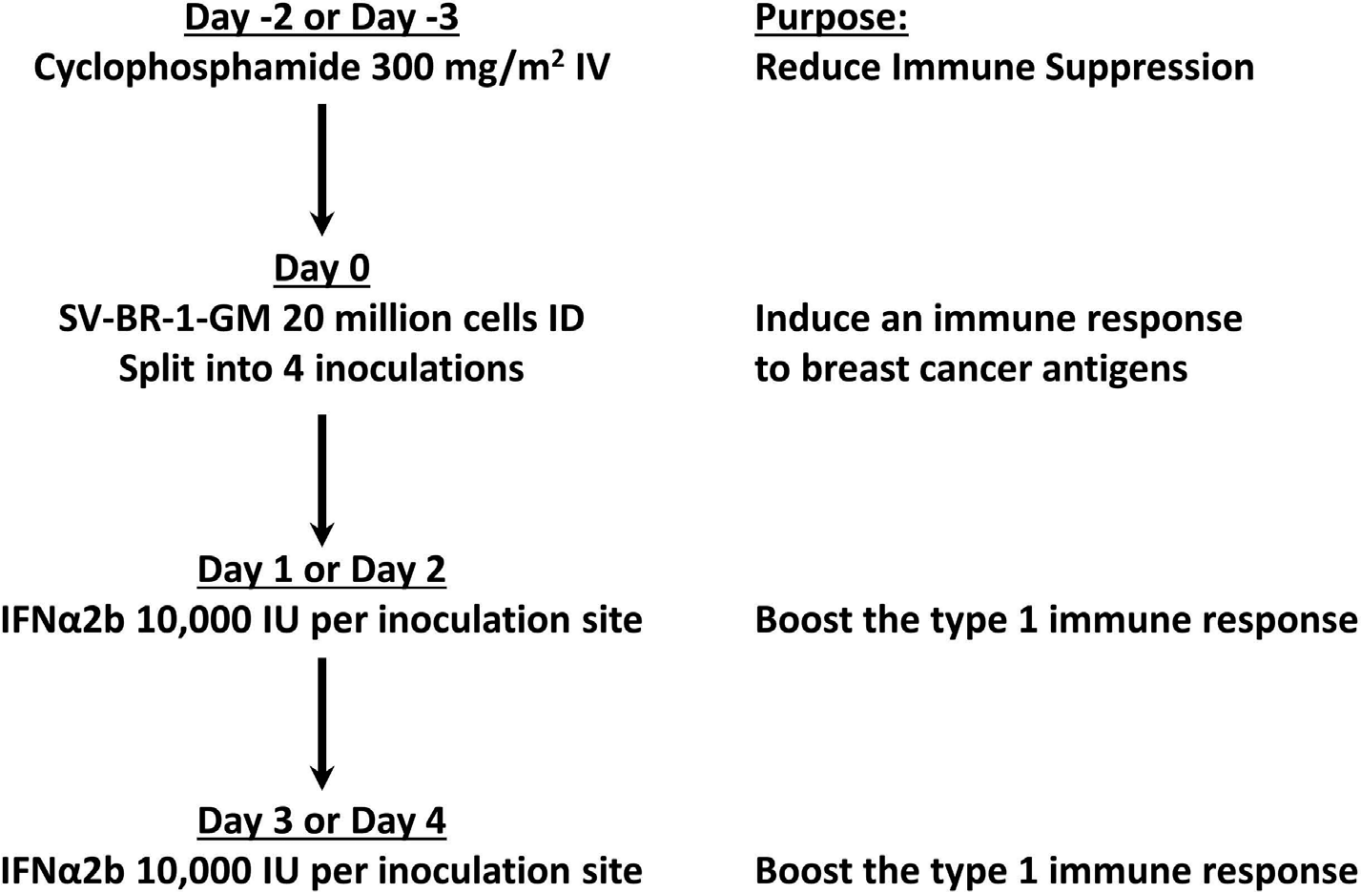
SV-BR-1-GM Treatment Regimen. The treatments given are shown in sequence with the rationale for the treatment shown on the right. IV = intravenously. ID = intradermally. IFNα2b = interferon-alpha 2b. IU = international units. Note that the SV-BR-1-GM cells were irradiated (200 Gy) before inoculation to prevent cell replication.

Each cycle of treatment consisted of the following (Fig. **1**):

- Cyclophosphamide 300 mg/m^2^ IV 48-72 hours before each SV-BR-1-GM inoculation.
- Intradermal inoculation of irradiated SV-BR-1-GM cells (20 million irradiated cells divided into 4 inoculation sites: anterior skin of the right and left thigh and over the right and left scapulae).
- 24-48 hours, and again 72-96 hours later, the patient was to receive 20,000 IU of IFN-α2b (Merck & Co., Inc., Whitehouse Station, NJ) in 0.1 mL (in Lactated Ringer's solution (LRS)) into each SV-BR-1-GM inoculation site^1^.

In addition, skin testing for immediate and delayed-type hypersensitivity (DTH) was performed in all patients using the non-genetically engineered parental cell line (SV-BR-1) prepared similarly as SV-BR-1-GM. Patients were injected intradermally with 1 x 10^6^ viable, irradiated SV-BR-1 breast tumor cells in a total volume of 0.1 mL LRS on the volar aspect of the forearm. Any evidence of immediate hypersensitivity would have aborted the therapeutic immunization with SV-BR-1-GM. The diameters of induration and erythema were recorded 24-48 hours later.

Patients received treatment cycles every 2 weeks for 3 inoculations during the first month, then monthly for 3 more treatments, provided no evidence of excess toxicity or evidence of new or enlarging tumor, for a total of 6 inoculations over a 4-month period.

## 3 RESULTS

### 3.1 Molecular Identity of SV-BR-1-GM

An immune signature proposed to contribute to SV-BR-1-GM’s mechanism of action has been identified by transcriptome analysis [21]. This includes the expression of specific HLA alleles which are proposed to present breast cancer antigens to immune cells. Here, we expand on these findings by quantifying the transcript levels of individual HLA alleles. Table **1** lists the *HLA-A*, *-B*, *-C*, *-DRB1*, *-DRB3*, *-DQB1*, and *-DPB1* alleles present in SV-BR-1 cells as determined by a DNA-based high-resolution method. We evaluated HLA allele expression in both unstimulated cells and cells stimulated with IFN-γ, an inflammatory cytokine that is likely present in the inflammatory milieu present at the SV-BR-1-GM inoculation sites in patients.

*RNA-seq to assess HLA allele expression levels:* RNA-seq was employed for this analysis. RNA was isolated from control and IFN-γ treated SV-BR-1 and SV-BR-1-GM cells. FASTQ files were processed using kallisto [36, 37] followed by normalization using sleuth [38]. We generated the kallisto index file using a FASTA file with cDNA sequences of the human genome assembly 38 (GRCh38). However, since the FASTA file contained sequences of HLA alleles absent in SV-BR-1 and SV-BR-1-GM cells (which could interfere, due to high sequence identity, with the estimation of the expression levels of the alleles actually present), we removed all *HLA-A*, *HLA-B*, *HLA-C*, *HLA-DRA, HLA-DRB1, HLA-DRB3, HLA-DRB4, HLA-DRB5*, *HLA-DQA1*, *HLA-DQB1*, *HLA-DPA1*, and *HLA-DPB1* transcript sequences in the FASTA file (Supplementary Data Sheet 1, Table **S1**). We then added the coding sequences (CDS) of only the alleles actually present in SV-BR-1 and SV-BR-1-GM cells (Table **1**). Pseudogene sequences were not removed. We only included the coding sequences because the untranslated region (UTR) sequences could not be determined for all of SV-BR-1-GM’s HLA alleles (Table **1**). Effects of UTR sequences in the index file are addressed below.

**Fig. (2):**
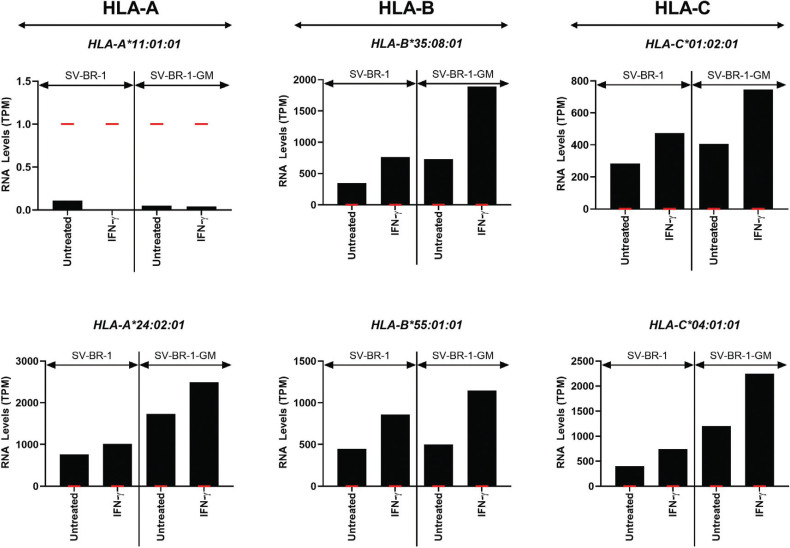
Class I HLA allele-specific mRNA expression in SV-BR-1 and SV-BR-1-GM. IFN-γ induces HLA class I expression. SV-BR-1 and SV-BR-1-GM cells were cultured in 6-well plates, with or without treatment with 50 ng/mL of IFN-γ for 16 hours, then subjected to gene expression analysis by RNA-seq. Shown are expression levels in transcript per million (TPM) as determined using kallisto [36, 37] followed by normalization using sleuth [38]. For the generation of the kallisto index file, HLA coding sequences without the 5’ and 3’ untranslated regions were used. Considering 1 TPM (red markers) as a threshold for expression, HLA-A*11:01:01 is not expressed. Note that the range for the TPM values shown on the Y-axes differs between different HLA alleles.

**Fig. (3):**
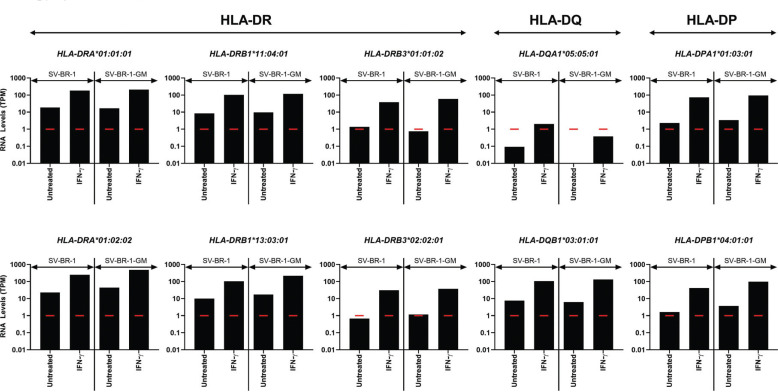
Class II HLA allele-specific mRNA expression in SV-BR-1 and SV-BR-1-GM. IFN-γ induces Class II HLA expression more substantially than Class I expression. SV-BR-1 and SV-BR-1-GM cells were cultured in 6-well plates, with or without treatment with 50 ng/mL of IFN-γ for 16 hours, then subjected to gene expression analysis by RNA-seq. Shown are expression levels in transcript per million (TPM) as determined using kallisto [36-37] followed by normalization using sleuth [38]. For the generation of the kallisto index file, HLA coding sequences without the 5’ and 3’ untranslated regions were used. Considering 1 TPM (red markers) as a threshold for expression, HLA-DRB3*01:01:02 and *HLA-DRB3:02:02:01* are at most marginally expressed in the absence of IFN-γ. Similarly, HLA-DQA1 is not (or only barely) expressed, even with IFN-γ, suggesting that HLA-DQ is not relevant in the SV-BR-1/SV-BR-1-GM system. In contrast, following treatment with IFN-γ, both HLA-DPA1*01:03:01 and HLA-DPB1*04:01:01 were expressed substantially, indicating a potential role in antigen-presentation in SV-BR-1 and SV-BR-1-GM cells. Note that the Y-axes are shown in log scale.

As shown in Fig. (**2**), all of SV-BR-1-GM’s Class I HLA alleles investigated were detected except HLA-A*11:01:01. Treatment with IFN-γ increased the Class I HLA alleles’ expression levels. Similarly, all *HLA-DRA* and *HLA-DRB1* alleles are expressed-albeit at lower levels than the Class I HLA alleles-while the *HLA-DRB3* levels fell below the cut-off for expression in the absence of IFN-γ. Of note, stimulation with IFN-γ induced a more substantial increase in expression for the Class II than for the Class I HLA alleles (Figs. **2** and **3**).

As shown in Fig. (**3**), especially if stimulated with IFN-γ, SV-BR-1-GM cells expressed both *HLA-DQB1* and *HLA-DPB1*. However, it is likely that despite being expressed, *HLA-DQB1* may not be relevant as only minute levels of *HLA-DQA1* were detected, suggesting that also on the cell surface, only marginal levels of functional HLA-DQ complexes are present. On the other hand, we did not obtain the *HLA-DRA*, *HLA-DQA1,* or *HLA-DPA1* allele specifications *via* a typing service, but rather attempted to identify the alleles by ourselves *via* comparisons between the sequences of known alleles and the RNA-seq reads. Since the reliability of this method with very low read counts is unknown, *HLA-DQA1* allele(s) present in SV-BR-1-GM cells may have been missed, and as such the contribution of HLA-DQ in the mechanism of action of SV-BR-1-GM underestimated. For HLA-DP, both *HLA-DPA1* and *HLA-DPB1* were expressed following IFN-γ treatment, but only very low levels were detected in the absence of IFN-γ (Fig. **3**).

To gauge the effect of the UTR sequences (or their absence) on the estimated transcript levels, *in silico* experiments were performed. The kallisto index file used to generate the data shown in Fig. (**2**), containing the coding sequences of the HLA alleles listed in Table **1**, was modified to also include corresponding UTR sequences as indicated in the IPD-IMGT/HLA database [43] for HLA-A*11:01:01:01, HLA-A*24:02:01:01, HLA-DRB3*01:01:02:02, and HLA-DRB3* 02:02:01:02. As shown in Supplementary Data Sheet **3**, Fig. (**S1**), for HLA-A*11:01:01:01, the presumptive expression levels remained below the cutoff used to define expression (TPM = 1) irrespective of whether or not UTR sequences were included. For HLA-A*24:02:01:01 and *HLA-DRB3*, the inclusion of the UTR sequences lowered the TPM values; however, left them well above the background levels (defined at 1 TPM) (Supplementary Data Sheet **3**, Figs. **S1** and **S2**).

Even though RNA-seq may allow gene expression analysis at the allele level, lack of (or inaccurate) sequence information can lead to misrepresentation of both the allele and gene expression levels. To understand whether the UTR sequences from the IPD-IMGT/HLA database [43] accurately reflect “actual” UTR sequences, we searched for transcripts in the Ensembl database (https://www.ense mbl.org/) with 100% sequence identity to the deduced transcript sequences from the IPD-IMGT/HLA database. None were found for HLA-A*11:01:01:01 and HLA-A*24:02:01:01 but one transcript (ENST00000383126.7) for HLA-DRB3*01:01:02:02 (HLA-DRB3*01:01:02:01) and two transcripts (ENST00000307 137.11 and ENST00 000426847.2) for HLA-DRB3*02:02:01: 02 were identified. All three transcripts encoded the full CDS plus UTR sequences of the respective *HLA-DRB3* alleles. With these *HLA-DRB3* Ensembl transcripts, the estimated expression levels (TPM values) for both HLA-DRB3*01: 01:02:02 and HLA-DRB3*02:02:01:02 were higher than for those from the IPD-IMGT/HLA database (Supplementary Data Sheet **3**, Fig. **S3**), suggesting that indeed, kallisto may not have captured all transcripts when using the UTRs from the IPD-IMGT/HLA database (Supplementary Data Sheet **3**, Figs. **S1** and **S2**).

**Fig. (4):**
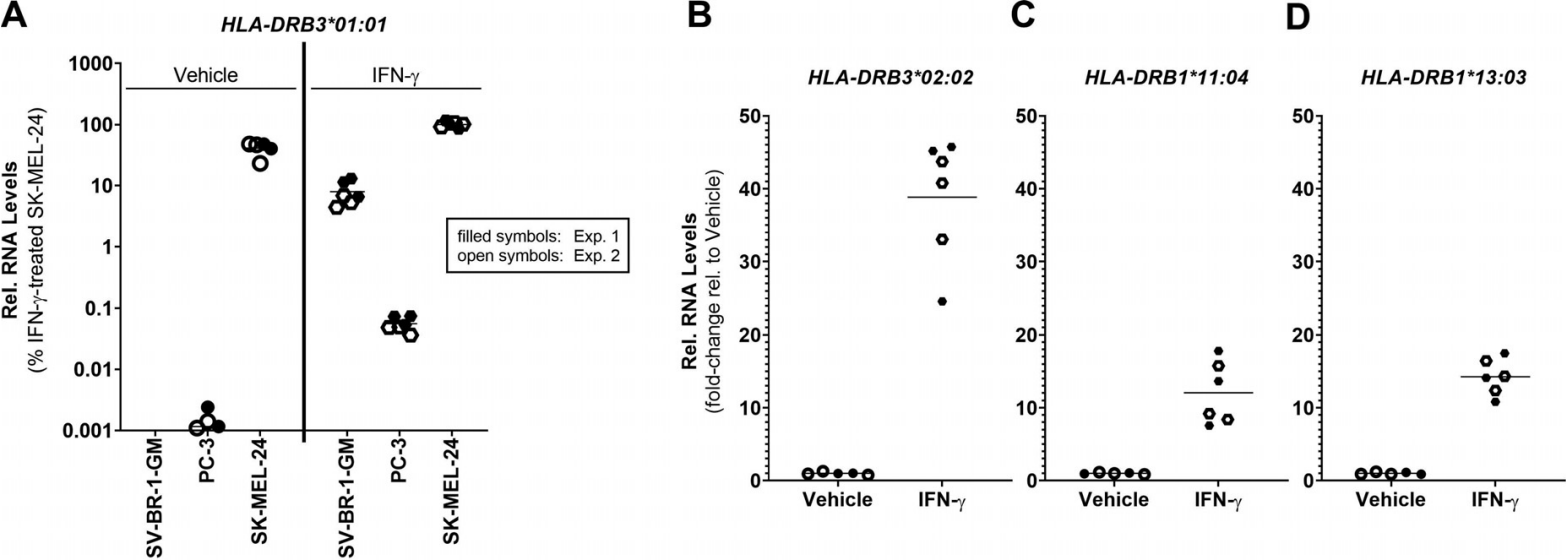
Validation of *HLA-DRB* mRNA expression by qRT-PCR. (**A**). In the absence of IFN-γ, HLA-DRB3*01:01 falls below 0.001% of the expression levels of those from SK-MEL-24 cells treated with IFN-γ (**B**), Fold-increase of HLA-DRB3*02:02 (**C**), HLA-DRB1*11:04 and (**D**) HLA-DRB1*13:03 expression upon IFN-γ treatment. The reference cell lines PC-3 and SK-MEL-24 could only be employed for HLA-DRB3*01:01 as the primer sets used to amplify HLA-DRB3*02:02, HLA-DRB1*11:04, and HLA-DRB1*13:03 alleles were not specific for the corresponding regions in the PC-3 and SK-MEL-24 cell lines. Shown are individual values from two experiments and arithmetic means (horizontal lines).

*qRT-PCR Analysis:* To confirm the expression of the *HLA-DRB1* and *HLA-DRB3* genes we established a SYBR Green-based quantitative RT-PCR (qRT-PCR) assay that distinguishes between the corresponding four *HLA-DRB* alleles present in SV-BR-1-GM. As demonstrated in Fig. (**4**), this assay essentially recapitulated the RNA-seq-based data Figs. (**2** and **3**). However, at least for HLA-DRB3*02:02, it appears somewhat more sensitive than the RNA-seq assay as the threshold cycle (Ct) values in the qRT-PCR assay for HLA-DRB3*02:02 were relatively low (data not shown) whereas the estimated transcript levels as assessed by RNA-seq were only around 1 TPM (Fig. **3**). For HLA-DRB3*01:01, after 40 cycles of PCR in the qRT-PCR assay, a strong band was apparent on a gel (Supplementary Data Sheet **3**, Fig. **S4**). However, the expression levels of HLA-DRB3*01:01 were below 0.001% of those from the melanoma cell line SK-MEL-24 (Fig. **4A**). As seen using RNA-seq (Figs. **2** and **3**), also with qRT-PCR we have observed that IFN-γ induced the expression of each of the *HLA-DRB* alleles assessed substantially (HLA-DRB3*01:01, (Fig. **4A**); HLA-DRB3*02:02, (Fig. **4B**); HLA-DRB1*11:04, (Fig. **4C**); HLA-DRB1*13:03, (Fig. **4D**). Note that the reference cell lines PC-3 and SK-MEL-24 could only be employed for HLA-DRB3*01:01 as the primer sets used to amplify HLA-DRB3*02:02, HLA-DRB1*11:04, and HLA-DRB1*13:03 alleles were not specific for the corresponding regions in the PC-3 and SK-MEL-24 cell lines.

**Fig. (5):**
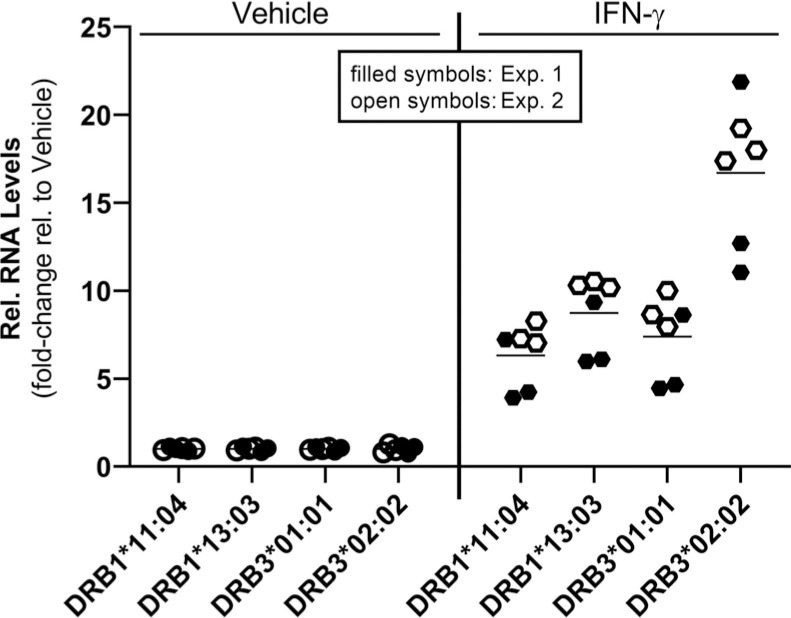
Effect of IFN-γ on *HLA-DRB* expression in irradiated SV-BR-1-GM cells. IFN-γ induced the expression of all 4 *HLA-DRB* alleles in irradiated SV-BR-1-GM cells. SV-BR-1-GM cells were irradiated with 100 Gy, cryopreserved, thawed, seeded, and then treated with 50 ng/ml of IFN-γ for 24 hours. Fold expression, relative to vehicle treatment, is shown. In contrast to non-irradiated SV-BR-1-GM cells, the irradiated then cryopreserved cells used did not adhere to the culture wells for the duration of the experiment. Shown are individual values from two experiments and arithmetic means (horizontal lines).

*HLA-DRB expression in irradiated SV-BR-1-GM cells:* As SV-BR-1-GM is inoculated into patients only following irradiation (to render the cells replication-incompetent) we sought to measure *HLA-DRB* expression in SV-BR-1-GM cells subjected to 100 Gy (10,000 Rad) of radiation as previously described (21). With Ct values of < 29, transcripts of both *HLA-DRB1* and both *HLA-DRB3* alleles could be readily detected by PCR even without IFN-γ stimulation (data not shown). Treatment with IFN-γ upregulated these basal expression levels (Fig. **5**), even though the extent of upregulation appears less substantial compared to the nonirradiated cells (Fig. **4**).

*Proteomic Analysis:* Expression of some of the HLA alleles was confirmed at the protein level by analyzing the proteome of SV-BR-1 employing a liquid chromatography (LC)-mass spectrometry (MS) based approach. As shown in Fig. (**6**), the presence of HLA-A*24:02, HLA-B*35:08, HLA-B*55:01, HLA-C*01:02, HLA-C*04:01, HLA-DRA, and HLA-DRB1 peptides was measured with high confidence (q < 0.001), confirming the corresponding RNA-based data (Supplementary Data Sheet **4**).

**Fig. (7):**
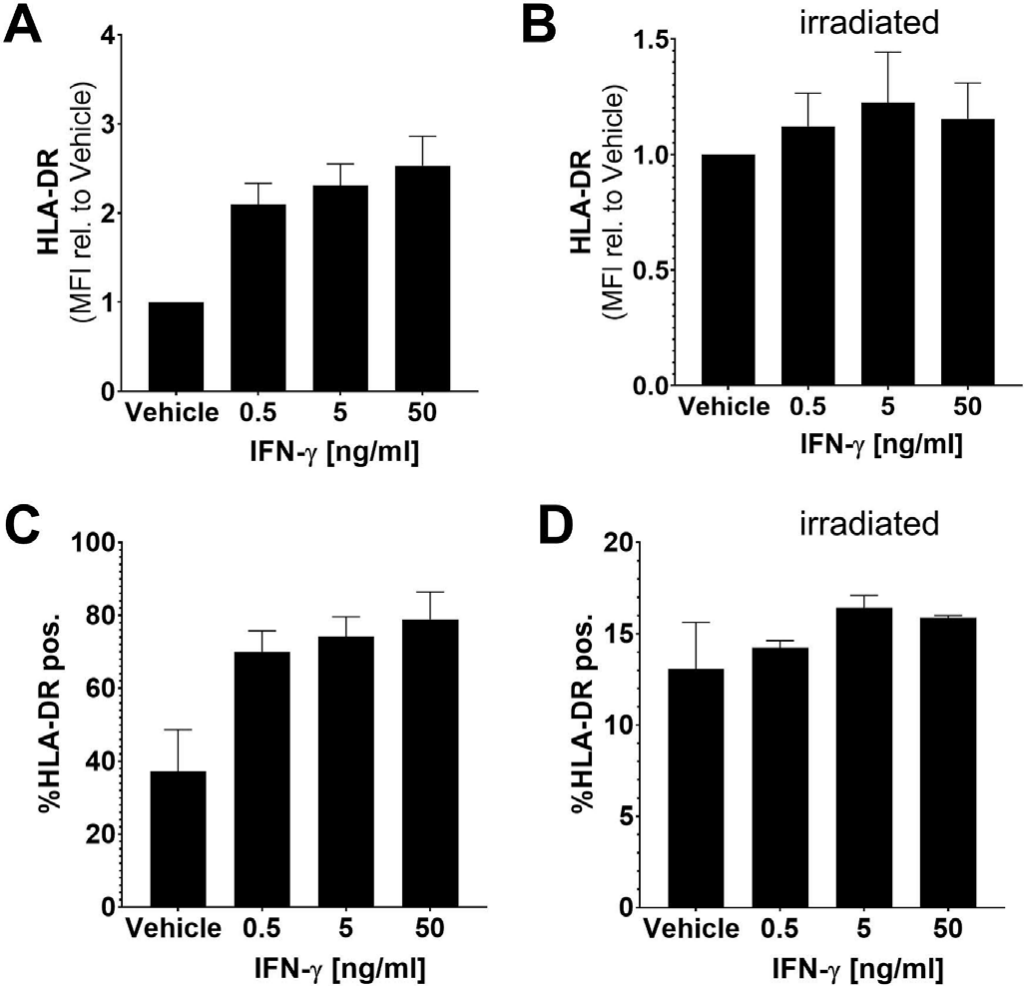
HLA-DR cell surface expression on SV-BR-1-GM cells. IFN-γ up-regulated HLA-DR cell surface expression in non-irradiated but not in irradiated SV-BR-1-GM cells. (**A**) Shown are vehicle-normalized mean fluorescence intensity (MFI) values in non-irradiated and (**B**) irradiated (**C**) SV-BR-1-GM cells or percent HLA-DR positive cells for non-irradiated and (**D**) irradiated SV-BR-1-GM cells.

*Flow Cytometry Analysis:* To confirm cell surface expression of Class II HLA (HLA-DR), we measured the HLA-DR levels using an antibody (clone L243) recognizing HLA-DR when the HLA-DR α and β chains are in complex [44]. As demonstrated in Fig. (**7**) and consistent with the RNA-based analyses (Figs. **3-5**), IFN-γ upregulated HLA-DR cell surface expression. As reported previously [21], irradiated SV-BR-1-GM cells expressed substantially lower levels of cell surface HLA-DR than nonirradiated cells. They were also not as responsive to IFN-γ stimulation as nonirradiated cells (Fig. **7**).

*Molecular grading of SV-BR-1-GM:* Histologically, breast cancer is typically divided into well-differentiated (grade 1), moderately differentiated (grade 2), and poorly differentiated (grade 3) as per the Nottingham (Elston-Ellis) modification of the Scarff-Bloom-Richardson grading system [45]. As noted above, SV-BR-1 (the parent cell line of SV-BR-1-GM) was derived from a tumor specimen from a patient with grade 2 breast cancer. Breast tumors may also be categorized as Luminal A, Luminal B (HER2+ and HER2-), HER2+ (often “HER2-enriched”), and ER-/PR-/HER2- (“triple-negative”) subtypes, with, in general, progressively worse prognosis in this order [46-48]. Furthermore, the triple-negative group can be divided into at least Basal, Claudin-low, MBC (metaplastic breast cancer), and interferon-rich groups [46, 49]. Similarly, breast cancer cell lines can be classified as Luminal, Luminal-HER2+, ER-/HER2+, and Basal (triple-negative; further differentiated into Basal A and B), with, in general, progressively more aggressive phenotypes predicted in this order [46, 50].

We wanted to understand whether a gene signature distinguishing the more differentiated grade 1 or 2 tumors from poorly differentiated grade 3 tumors could serve as a tool to quantify the degree of differentiation of breast cancer cell lines, especially of SV-BR-1-GM. Whereas others also describe gene signatures for distinguishing breast tumor grade [51, 52], to our knowledge, only our analysis described here is based on more than 1500 tumor samples. Using GENEVESTIGATOR^®^ (NEBION AG, Zurich, Switzerland), a manually curated database and analysis platform for publicly available transcriptomic data sets [41, 42], we conducted a meta-analysis of 7 microarray studies with a total of 1607 samples with grading information. Each microarray study was individually queried for genes expressed either ≥2 times *higher* or ≥2 times *lower* in grade 3 tumors compared to grade 1 or 2 tumors. Genes present in the signatures of at least 5 out of the 7 studies were included in the “consensus signature” CS55 (Supplementary Data Sheet **2**, Table **S2**).

From this 55-gene consensus signature, 54 genes (CS54 signature) were represented in a normalized data set used previously (to generate Fig. (**8**) in [21]). Of note, we could not verify whether for all 7 studies the grading was conducted according to the same method, but given that the modified SBR criteria were introduced about 3 decades ago [45] it is likely that those were followed, even if this was not explicitly stated as such and descriptions, for example, may have only included “SBR” (without “modified”). Nevertheless, even if different methods were employed, our approach is based on a gene signature present in multiple data sets and as such is expected to provide a means for discriminating tumors with favorable *vs.* poor prognoses based on histological criteria regardless of the exact grading protocol followed.

To estimate the tumor grade represented by the SV-BR-1-GM cell line, we developed a score we refer to as Relative Molecular Grade (RMG) taking the expression levels of each of the CS54 signature genes into account (weighted according to the fold-changes in the meta-analysis) and indicating the relative degrees of “differentiation” among the samples analyzed. As shown in Fig. (**8**), with an RMG of 58.5, SV-BR-1-GM is most similar to the MDA-MB-468 cell line (RMG of 51.9), which was classified as Basal A [46, 53]. Given that overall, the Basal A subtype is predicted to be less aggressive than the Basal B subtype [46, 50] and grade 1 and 2 breast cancers tend to have a better prognosis and as such can be considered less aggressive than grade 3 breast cancers [45], it is tempting to speculate that with its association with the Basal A subtype, SV-BR-1-GM has at least partially retained the grade 2 character of the patient’s breast cancer from which the SV-BR-1-GM cell line was derived.

### 3.2 Clinical Study

Four patients were treated under this protocol with the patient characteristics noted in Table **2**. All patients were post-menopausal white women aged between 58.7 and 73 years. All were diagnosed with breast cancer except A003 (HER2+ ovarian cancer). Likewise, all patients had ECOG performance status 1 except patient B001 (ECOG 2). All patients had received at least one prior systemic therapy. Additional information on the clinical study is provided in Supplementary Data Sheet **5**. Patients A002 and B001 matched with SV-BR-1-GM at *HLA-DQB1* and *HLA-DPB1*, respectively (Table **1**).

*The extent of Exposure:* Patients received 3 cycles at 2-week intervals, then 3 cycles at 1-month intervals. All patients underwent extensive baseline imaging and were restaged 1 month after the third and last inoculation (*i.e*., 2 and 5 months after the initial inoculation). The median number of cycles was 5 (range 4-6). Patients remained on study a median of 95 days (range 58-117 days, Table **3**). One patient who has been previously described and is here referred to as “Patient A002” completed treatment, but relapsed 106 days after her last inoculation [30]. After obtaining permission from the FDA, the patient was treated off-protocol for 13 additional cycles of therapy, the last eight with the addition of vinorelbine tartrate to a total of 229 additional days on treatment (Table **3**).

*Safety:* The treatment was generally safe and well-tolerated in this small study. There were no deaths during the study. There were four grade 3 serious adverse events (SAEs) reported in 3 patients with no grade 4 events with one judged related to the study drug: Grade 3 transient urticaria in patient A001 who responded to antihistamines. There were 29 adverse events observed during the study (3 observed twice in the same patient) with almost all mild or moderate in severity (Table **4**) except grade 3 itch, rash, colitis, and back pain. The only adverse events occurring in more than one patient were back pain, constipation, itch, and fatigue.

*Immune Responses:* DTH to the parental cell line, SV-BR-1, was evaluated at each cycle of treatment. The therapeutic inoculation sites were similarly evaluated for induration and erythema. As shown in Fig. (**9**), all patients developed evidence of DTH to SV-BR-1 and/or SV-BR-1-GM, indicating a robust cellular immune response.

IgG antibody responses to SV-BR-1, identified by flow cytometry, are shown in Fig. (**10**). Three of the 4 patients developed humoral immune responses to SV-BR-1, with the most marked response seen for Patient A002, who also had objective evidence of tumor regression (see below). Antibody levels peaked at Cycles 5 and 6 and then declined. Patient A001 experienced a marked decline in the anti-SV-BR-1 IgG antibody levels upon immunization compared to baseline. The significance of this observation is unknown.

*Clinical Responses:* Median time to tumor progression was 144 days (range 64 - 223 days) for the initial round of treatment. Overall survival was more than 33 months in all patients except B001 (7 months). One patient (A002) had a second and third round (Series 2 and 3) of treatment after having relapsed 106 days after her last inoculation (Table **3**) [30]. Data on survival and clinical responses of all patients are also shown in Table **3**.

Patient A002 matched SV-BR-1-GM at the *HLA-DRB1*, *HLA-DRB3*, and *HLA-DQB1* loci (Table **1**).This finding supports a postulated antigen-presentation role of SV-BR-1-GM [21]. One patient (A001) matched SV-BR-1-GM at a Class I but not a Class II HLA locus (HLA-A*24:02), and another Patient (B001) matched SV-BR-1-GM at both a Class I and a Class II locus (HLA-C*04:01 and HLA-DPB1*04:01:01)(Table **1**).

Patient A002, described previously [30], had metastatic breast cancer affecting the breast, lung, soft tissue, and bones, resistant to chemotherapeutic agents, radiation therapy, and anti-hormonal therapy. At two months she had a complete regression of a previously enlarging pulmonary lesion and near-complete regression of the breast lesions at 5 months (Supplementary Data Sheet **3**, Fig. (**S5**), [30]). Three and a half months after the last inoculation, PET, CT, and MRI studies identified multiple areas of recurrence, including lung, right breast, several brain metastases, lesions in the mediastinum, and probable involvement in the liver. Permission was obtained from the FDA to reinitiate SV-BR-1-GM treatments with cycles every 2 weeks. Following three additional cycles, marked regression of multiple brain and breast lesions was noted, and improvement in the liver and chest lesions [30]. Although she later relapsed, the CNS metastases remained in remission for 26 months until expiration.

### 3.3 Patent Review

This and our previous [21] analyses of patient HLAs *vs.* clinical response data have identified a relationship between the presence of certain HLA molecules and response to SV-BR-1-GM, suggesting that lack of HLAs in certain patients may be a prohibitive factor for maximum benefit from the treatment. To overcome such potential limitations, we are developing cell lines similar to SV-BR-1-GM engineered with different exogenous HLA alleles. The four HLA-diverse cell lines in development are designed to overexpress a total of seven HLA-A and eight HLA-DRB3/4/5 alleles. These are expected to cover the vast majority of Americans with an HLA class I and II double-match, taking multiple races and ethnic groups into consideration. The work has been addressed by our PCT patent application (publication number: WO2017147 600A1; “Whole-cell cancer vaccines and methods for selection thereof”) [22]. The following paragraphs outline the differences between several key patents/patent applications and WO2017147600A1.

WO2017147600A1 [22] and its derivatives, National stage applications such as EP3419657A4 (Europe; patent pending) [31] and JP6901505B2 (Japan; granted patent) [32], claim, among others, SV-BR-1 cells engineered to overexpress HLA alleles in combination with GM-CSF and IFN-α, cytokines with roles in enhancing immune responses.

EP0569678A2 [23] and US5750102A [24] (“Double transfectants of the MHC genes as cellular vaccines for immune prevention of tumor metastasis”) are similar to WO20171-47600A1 [22] in that claims relate to the overexpression of HLA alleles in tumor cells but co-expression of cytokines is not claimed.

US6149905A (“Tumor cells with increased immunogenicity and uses therefor”) [25], US201001195 37A1 (“Tumor Vaccine”) [26], and US7807186B2 (“Tumor cells from immune-privileged sites as base cells for cell-based cancer vaccines” [27]) go a step further than EP0569678A2 [23] and US5750102A [24] in that they focus on B7 co-stimulatory molecules and claim those in combination with MHC molecules. They also do not claim co-expression of HLA molecules and cytokines.

WO2012156969A1 (“Allogeneic tumor cell vaccination”) [28] claims allogeneic cell lines engineered to express HLA alleles and/or co-stimulatory molecules for T cells such as 4-1BB Ligand. The patent application claims co-expression of HLA molecules and melanoma antigens but not of HLA molecules and cytokines.

US7674456B2 (“Breast cancer cell lines and uses thereof”) is the pre-cursor patent to Bria-OTS, claiming compositions comprising SV-BR-1 cells [29].

Taken together, whereas other investigators have also claimed cancer cells overexpressing HLA molecules, WO20-17147600A1 [22] (and its derivatives) additionally claims ectopic co-expression of GM-CSF and/or IFN-α.

## 4 DISCUSSION

This report of a very small number of patients nonetheless produces a considerable number of notable observations. The responses of Patient A002 seem to be in disagreement with the commonly accepted notion that cellular immunization is unlikely to be effective against the macroscopic disease. Unraveling the specific mechanisms of action is the major focus of our research. Notably, the cell line used for immunization and Patient A002 matched at several Class II HLA alleles. Molecular characterization of the SV-BR-1-GM cell line, both its functionality and its putative antigens, provides additional potential biomarkers. Clinical trials such as this one permit documentation and analysis of immunological responses. Our results should inform testable immunization strategies going forward, particularly in selecting the patients most likely to benefit and with the design of more potent cellular immunotherapies.

### 4.1 Clinical Correlates

The contribution of histocompatibility alleles to clinical responses may involve multiple mechanisms. As such, they could present tumor antigens to CD4+ T_H_ cells by Class II HLA molecules. Also, they could stimulate cytotoxic CD8+ cells with tumor antigens presented by Class I HLA molecules. For the Class II HLA matches, most were with *HLA-DRB3* rather than *HLA-DRB1* (data not shown), which may be mostly explained by the higher degree of polymorphism at the *HLA-DRB1* compared to the *HLA-DRB3* locus. However, as shown in this study, *HLA-DRB3* is expressed only at low levels in SV-BR-1-GM cells in the absence of IFN-γ, which may raise questions regarding the relevance of *HLA-DRB3* for SV-BR-1-GM’s mechanism of action. Along the same lines, A002 was the only subject among those with tumor regressions with an *HLA-DRB1* (in addition to an *HLA-DRB3*) allele match *and* also the patient who experienced by far the strongest anti-tumor, clinical responses in this study.

Note that the questionable relevance of *HLA-DRB3* is-or appears to be-in stark contrast to our previous findings, where we demonstrated a functional contribution of *HLA-DRB3* to SV-BR-1-GM’s activity [21]. Specifically, we showed seemingly substantial expression levels of the *HLA-DRB3* alleles present in SV-BR-1-GM using nCounter (NanoString Technologies, Inc.; Seattle, WA, USA) probes and demonstrated that SV-BR-1-GM is capable of stimulating an *HLA-DRB3*-restricted T cell clone in an antigen-specific manner [21]. Potentially, the nCounter probes used to measure *HLA-DRB3* cross-reacted with SV-BR-1-GM’s *HLA-DRB1* alleles (sequence identity 91% and 93%, data not shown). Nevertheless, in the functional experiment, IFN-γ levels were upregulated after coculturing a T cell clone specific for a yellow fever virus (YFV) peptide in an HLA-DRB3* 01:01 context with YFV peptide-pulsed SV-BR-1-GM cells, providing evidence that HLA-DRB3*01:01 is functional in SV-BR-1-GM cells [21]. There are several possible explanations for this discrepancy. One is that the cutoff for RNA expression we used here (1 TPM) was arbitrary and may be too high. Notable, both RNA stability and protein stability vary for different gene products, and it is possible that long-lived RNA could result in numerous proteins being translated despite low RNA levels, and the protein product may also persist and maintain relatively high levels. However, it can also be imagined that in the *in vitro* experiment, the high background levels of IFN-γ noted with the T cell clone in the absence of SV-BR-1-GM (data not shown) were sufficient to promote upregulation of HLA-DRB3*01:01, which in turn further stimulated the T cell clone. As noted in Fig. (**5**), even irradiated SV-BR-1-GM cells are capable of upregulating *HLA-DRB3* expression in response to IFN-γ. The key question then would be whether *in situ*, in the inoculation sites, an inflammatory milieu occurs with sufficiently high levels of IFN-γ to induce HLA-DRB3 expression. Another possibility is that (a) HLA-DR molecule(s) expressed by SV-BR-1-GM (such as *HLA-DRB1*) is/are able to function similarly to HLA-DRB3*01:01. Whichever of these explanations, if any, is correct, the notable partial/near-complete and complete tumor regressions seen with Subject A002 were unique [30]. Given that A002 HLA matched SV-BR-1-GM at both *HLA-DRB1* and *HLA-DRB3* loci, it seems reasonable to speculate that patients with *HLA-DR* matches are the most likely to benefit from SV-BR-1-GM immunotherapy. This may be important for other whole-cell immunotherapies as well [54-58].

## CONCLUSION

Here, we have presented details of a clinical study whose original design exploited several synergistic processes. We utilized SV-BR-1-GM as a source of antigens and as a source for the local release of GM-CSF; we subsequently identified expression of Class II HLA alleles, possibly active in antigen presentation and activation of CD4+ (as opposed to CD8+) lymphocytes.

We utilized low-dose cyclophosphamide and intra-inoculation injection of low-dose interferon-alpha, based on the understanding of their actions, a design supported by later publications. Among the beneficial effects of this regimen, we observed prompt regression of breast cancer metastases occurring consistently in multiple different sites in a special responder. Moreover, we showed comparable responses again after relapse and retreatment. The regimen produced a durable complete regression of CNS metastases. While the data here may be considered anecdotal, we feel that the possibly unique findings are not easily dismissed and certainly encourage vigorous further study. These effects of this regimen on advanced metastatic breast cancer in a heavily-pretreated subject counter many widely-accepted assumptions, and offer many novel testable hypotheses regarding the importance of Class II HLA expression on whole-cell immunotherapies.

The findings described here strongly encourage our efforts to engineer cancer cell lines to overexpress HLA molecules. We envision this pending work may improve patient selection, and also might enhance the immune response to cancer cell lines with low endogenous HLA expression levels. WO2017147600A1 [22] and its National stage derivatives support translating these findings into the clinic.

Sources of Bias: This was a single-arm open-label study conducted under a physician-associated Investigational New Drug Application. As such, the results are very preliminary and need to be confirmed in additional studies.

## Figures and Tables

**Fig. (6):**
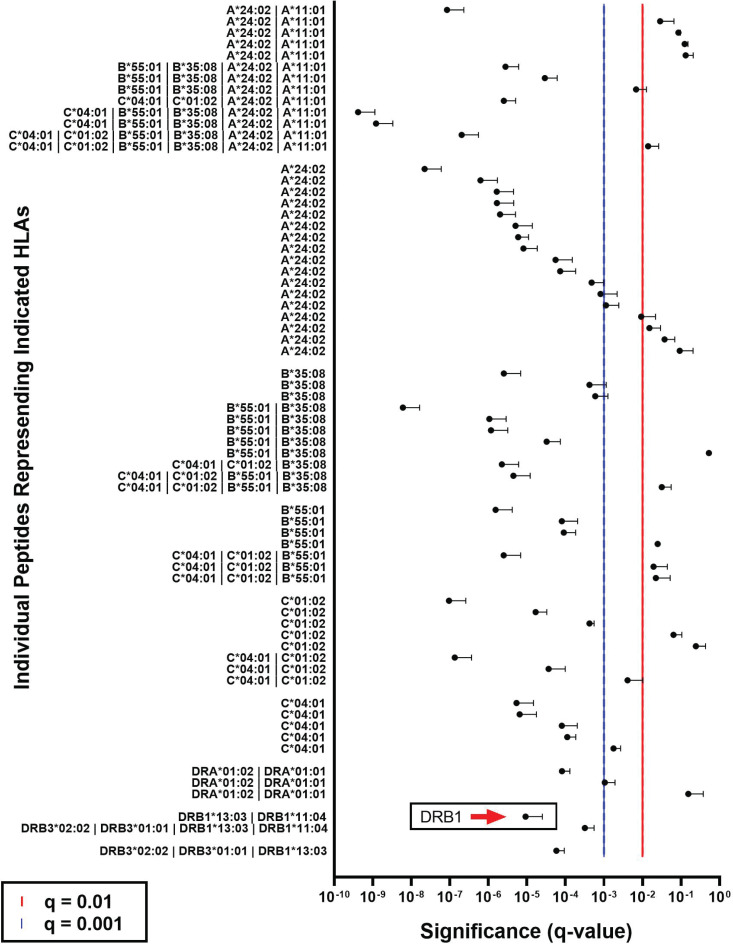
HLA protein expression in SV-BR-1. SV-BR-1 cells were detached using TrypLE Express, then cell pellets were frozen and submitted to Biognosys AG (Schlieren, Switzerland) for liquid chromatography (LC)-mass spectrometry (MS) based protein screen. The presence of HLA-A*24:02, HLA-B*35:08, HLA-B*55:01, HLA-C*01:02, HLA-C*04:01, HLA-DRA, and HLA-DRB1 (arrow) peptides was measured with high confidence (q < 0.001). Shown are arithmetic means and standard deviations (SDs) (one direction only) of the q values from triplicate SV-BR-1 cultures. Each symbol represents a unique peptide, which in turn represents at least one HLA allele present in SV-BR-1 cells. The lower the q value, the more likely the HLA protein represented by the peptide is expressed in SV-BR-1 cells. Red and blue lines indicate q = 0.01 and q = 0.001, respectively. The sequences of the peptides are shown in Supplementary Data Sheet **4**, Table **S4.**

**Fig. (8):**
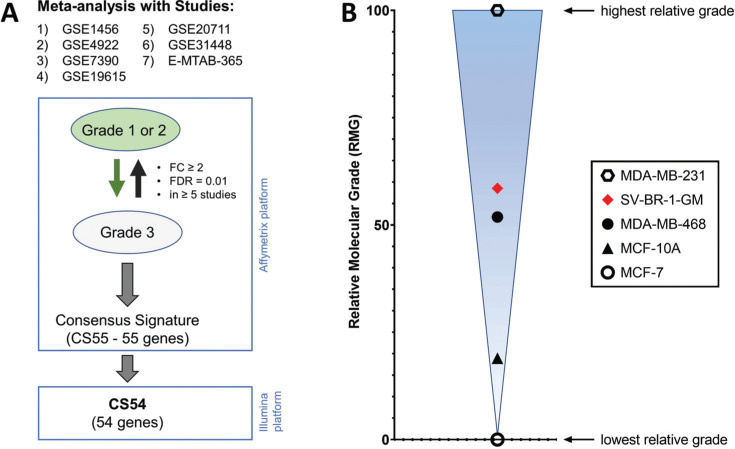
Molecular signature distinguishing grade 1 and 2 from grade 3 breast tumors. (**A**) Meta-analysis to identify a gene signature distinguishing grades 1 and 2 from grade 3 breast tumors. Using GENEVESTIGATOR^®^ (NEBION AG, Zurich, Switzerland) [41, 42], seven (7) breast cancer studies run on Affymetrix platforms with grading information were identified and then individually queried for genes expressed either ≥2 times *higher* or ≥2 times *lower* (FC ≥ 2) in grade 3 tumors compared to grade 1 or 2 tumors. Genes present in the signatures of at least 5 out of the 7 studies were included in the “consensus signature” CS55 (Supplementary Data Sheet **2**, Table **S2**). From this 55-gene consensus signature, 54 genes (CS54 signature) were represented in a normalized data set used previously (to generate Fig. (**8**) in [21]). A false discovery rate (FDR) of 0.01 was chosen. (**B**) To estimate the tumor grade represented by SV-BR-1-GM cells, we developed a score referred to as Relative Molecular Grade (RMG) taking the expression levels of each of the CS54 signature genes into account, weighted according to the fold-changes in the meta-analysis. With a similar RMG, SV-BR-1-GM cells most closely resemble the triple-negative MDA-MB-468 cell line, representing the Basal A subtype [46, 53]. FC, fold-change.

**Fig. (9):**
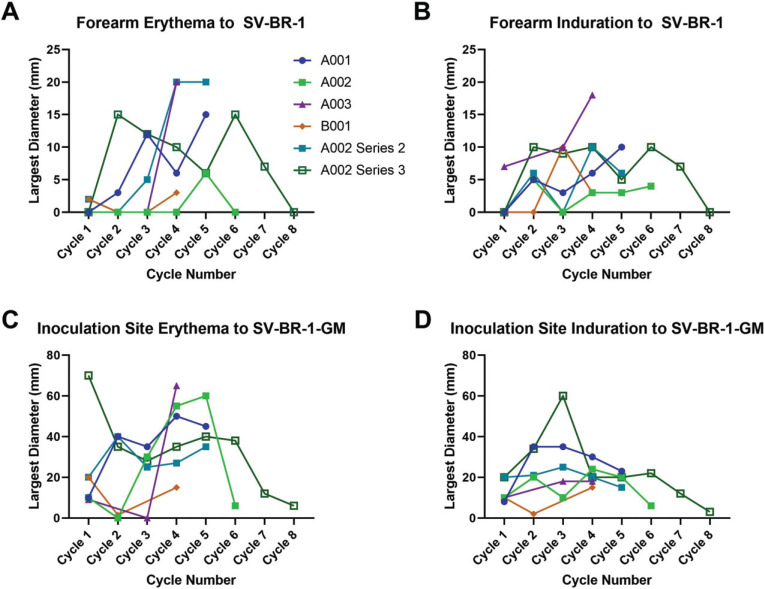
DTH following inoculation with SV-BR-1 and SV-BR-1-GM. 1 million irradiated SV-BR-1 cells were intradermally inoculated on the volar aspect of the forearm to assess immediate hypersensitivity to the cells (skin test). The absence of immediate hypersensitivity is a requirement for continuing with the experimental treatment. (**A**) Erythema and (**B**) induration responses 24-48 hours after the injections at the skin test sites with the largest diameters shown. Similarly, delayed-type hypersensitivity (DTH) was evaluated for the therapeutic intradermal inoculation sites (5 million irradiated SV-BR-1-GM cells per site, for a total of 4 sites: 2 sites in thighs and 2 sites over the scapulae), with the (**C**) erythema and (**D**) induration responses noted 24-48 hours later. The largest reaction of the four inoculation sites was plotted. Patient A002 had 2 additional series off study: Series 2 was similar to the first series (on-study). Series 3 was in combination with vinorelbine tartrate (Navelbine). The first SV-BR-1-GM inoculation of Series 3 occurred 2 weeks after the last SV-BR-1-GM inoculation of Series 2.

**Fig. (10):**
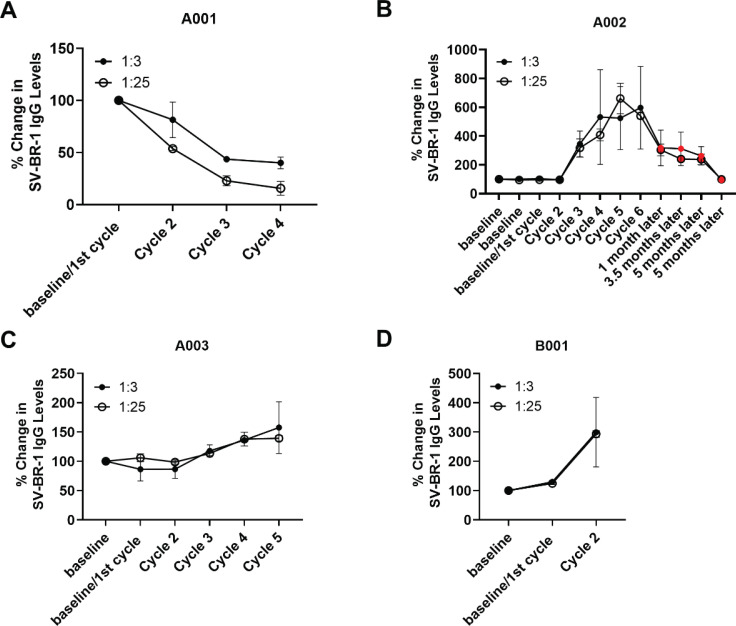
Anti-SV-BR-1 IgG levels in patient sera. Whereas Patients A002, A003, and B001 responded to the SV-BR-1-GM regimen with increased anti-SV-BR-1 antibody levels, Patient A001’s levels decreased. SV-BR-1 cells were incubated with 1:3 or 1:25 diluted patient sera then stained with fluorescently labeled anti-human IgG and analyzed by flow cytometry, (**A**), A001 (**B**), A002 (**C**), A003 (**D**), B001. Shown are arithmetic means with SDs from 2 flow cytometry runs with the same sera samples. Note that for A002 (**B**), 4 time points after completion of Series 1 (6 cycles) are shown. The first inoculation of Series II occurred soon after the last time point shown (5 months after cycle 6).

**Table 1:** HLA alleles in SV-BR-1-GM. HLA alleles as determined for SV-BR-1. The coding sequences (CDS sequences), defined by the first 3 sets of digits, of all of the indicated HLA alleles were identified. For a subset of the alleles, additional information on untranslated regions (4^th^ set of digits) was obtained. *HLA-DRA*, *HLA-DQA1* and *HLA-DPA1* alleles were deduced from RNA-seq data. Alleles highlighted in bold in the columns for the individual Subjects (A001, A002, A003, B001) denote alleles that are at the CDS level (first 3 sets of digits) both present *and* expressed in SV-BR-1-GM cells, at least following stimulation with IFN-γ. All four patients (also) had group matches: Subject A001 and A002 at *HLA-DRB1* (DRB1*13), A002 at *HLA-DPB1* (DPB1*04:02), A003 at *HLA-DQB1* (DQB1*03) and at *HLA-DPB1* (DPB1*04), and B001 at *HLA-B* (B*35).

**-**	**SV-BR-1-GM**	**A001**	**A002**	**A003**	**B001**
**HLA-A**	*11:01:01:01*	*02:01:01*	*02:01:01*	*02:01:01*	*11:01:01:01*
	*24:02:01:01*	***24:02:01:01***	*11:01:01:01*	*03:01:01:01*	*11:01:01:01*
**HLA-B**	*35:08:01:01*	*13:02:01:01*	*18:03*	*07:02:01*	*35:01:01*
	*55:01:01*	*41:01:01*	*44:02:01:01*	*13:02:01:01*	*40:01:02*
**HLA-C**	*01:02:01*	*06:02:01*	*05:01:01*	*06:02:01*	*03:04:01*
	*04:01:01:06*	*17:01:01:05*	*07:01:01*	*07:02:01*	***04:01:01:05***
**HLA-DRA**	*01:01:01* *01:02:02*	N.D.	N.D.	N.D.	N.D.
**HLA-DRB1**	*11:04:01*	*07:01:01*	***11:04:01***	*07:01:01*	*07:01:01*
	*13:03:01*	*13:02:01:02*	*13:01:01*	*07:01:01*	*15:01:01*
**HLA-DRB3**	*01:01:02:02*	*03:01:01*	***02:02:01:02***	*-*	*-*
	*02:02:01:02*	*-*	***02:02:01:02***	*-*	*-*
**HLA-DQA1**	*05:05:01*	N.D.	N.D.	N.D.	N.D.
**HLA-DQB1**	*03:01:01:03*	*02:02:01*	***03:01:01:03***	*02:02:01*	*02:02:01*
	*03:01:01*	*06:04:01*	*06:03:01*	*03:03:02:01*	*06:02:01:01*
**HLA-DPA1**	*01:03:01*	N.D.	N.D.	N.D.	N.D.
**HLA-DPB1**	*04:01:01*	*02:01:02*	*02:01:02*	*04:02:01*	***04:01:01***
	*04:01:01*	*04:02:01*	*04:02:01*	*19:01:01*	*17:01:01:01*

**NOTE:** HLA = human leukocyte antigen; N.D = not determined or not reported.

**Table 2:** Baseline and demography characteristics of patients treated with the SV-BR-1-GM regimen.

**Subject ID**	**Cancer diagnosis**	**Years to 1st met.**	**Age**	**Ethnicity** **Race**	**ECOG**	**TNM stage**	Grade*	**Sites of disease**	**ER/ PR**	**Her2/** **neu**
A001	Breast	3.5	73	CaucasianWhite	1	T1N0M0	3	Chest wall, Axilla	Neg/ Neg	+
A002	Breast	0	58.7	Caucasian White	1	T2N3M1	2	Breast, Lung	ER+/ PR+	-
A003	Ovarian	24	72.2	Caucasian White	1	T4	N/A	Pelvis	NA	+
B001	Breast	0	60.8	Caucasian White	2	T2NXM1	2	Rib cage, Humerus, Hip, Femur	ER+/ PR+	-

**NOTE:** *Modified Scarff-Bloom-Richardson (SBR) Grade (23): 1 = well differentiated; 2 = moderately differentiated; 3 = poorly differentiated. Abbreviations: met., metastasis; ECOG, Eastern Cooperative Oncology Group; ER, estrogen receptor; PR, progesterone receptor.

**Table 3:** Treatment administered, time to tumor progression, and survival and clinical response.

**Patient ID**	**Number of Inoculations**	**Time on Treatment** (**Days**)	**Time to Tumor Progression** (**Days**)	**Dx to Rx** (**Years**)	**Survival** (**Months**)	**Tumor** **Regression**
A001	5	98	128	6	40.7	No
A002	6 and 13*	117 and 229*	223 and 160*	2	33.7	Yes
A003	5	91	159	33	35.6	No
B001	4	58	64	10	7.0	No

**NOTE:** *Second course of treatment, with 10 biweekly inoculations then 3 monthly inoculations; Dx = diagnosis; Rx = treatment.

**Table 4:** Number of patients with adverse events.

**Verbatim Term**	**Number of Patients Affected**	**Grade**
Back pain	3	1-3
Constipation	2	1-2
Itch	2	2-3
Fatigue	2	2
Anemia	1	2
Colitis	1	3
Coughing	1	2
Diarrhea	1	2
Diverticulosis	1	2
Fever	1	2
Hemorrhoids	1	2
Induration at inoculation sites	1	2
Mucus plug	1	1
Nausea	1	1
Positional vertigo	1	1
Rash	1	3
Redness	1	2
Slight cough	1	1
Urinate frequently	1	2
Weakness	1	2
Wheel and flare	1	2

## Data Availability

The RNA-seq raw data files, in FASTQ format, were submitted to the Sequence Read Archive (SRA) [54] and are available under SRA, BioProject accession PRJNA658521. The mass spectrometry proteomics data set was submitted to the ProteomeXchange Consortium *via* the PRIDE [55] partner repository with the dataset identifier PXD020304.

## Footnotes

1 Patient A001 received 2,000 IU interferon-α2b before a subsequent amendment to the protocol.
