# Supplementary material for: Regression of Breast Cancer Metastases Following Treatment with Irradiated SV-BR-1-GM, a GM-CSF Overexpressing Breast Cancer Cell Line: Intellectual Property and Immune Markers of Response
Source: Recent Pat Anticancer Drug Discov. 2023 Dec 28;18(2):224–40. doi: 10.2174/1574892817666220518123331 (PMC10009895; doi:10.2174/1574892817666220518123331)
Supplement: Supplementary file 1 [file PRA-18-224_SD1.zip › PRA-18-224_SD1/BMS-PRA-2021-94-SUPPLEMENTARY MATERIAL.pdf]

SUPPLEMENTARY MATERIAL

Regression of Breast Cancer Metastases Following Treatment with Irradiated SV-BR-1-GM, a GM-CSF Overexpressing Breast Cancer Cell Line: Intellectual Property and Immune Markers of Response

Charles L. Wiseman[1], Alexander Kharazi[4], Vivekananda G. Sunkari[1], Jacqueline L. Galeas[1], Vito Dozio[2], Hind Hashwah[3], Eva Macúchová[3], William V. Williams[1] and Markus D. Lacher[1,*]

[1]BriaCell Therapeutics Corporation, 2929 Arch Street, 3rd Floor, Philadelphia, PA 19104, USA; [2]Operations Department, Biognosys AG, Wagistrasse 21, 8952 Schlieren, Switzerland; [3]Sales and Marketing Nebion AG, Hohlstrasse 515, 8048 Zurich, Switzerland; [4]Immunotherapy Laboratory, St. Vincent Medical Center, Los Angeles, CA, USA

SUPPLEMENTARY CLINICAL TABLES

Table 1S. Study Hypothesis and Objectives.

Hypothesis: Inoculation of previously treated breast cancer and ovarian cancer patients with SV-BR-1-GM will have no serious toxicities

Objectives:

1.1 To evaluate the safety and feasibility of vaccine therapy for advanced breast cancer using a HER-2/neu positive allogeneic breast tumor cell line transfected with the GM-CSF gene, admixed with PEG/interferon alfa-2b (PEG-Intron), and inoculated by intradermal injection.
1.2 To evaluate clinical response, quality of life, time to progression and survival.
1.3 To evaluate immune responses to tumor vaccine antigens and, whenever possible, to autologous tumor antigens.

Table 2S. Setting.

Medical office of Charles Wiseman MD FACP

201 S Alvarado St

Los Angeles, CA 90057

Table 3S. Eligibility Criteria and Periods of Recruitment.

Key inclusion criteria

1. Aged 18 years or older
2. Histological confirmation of stage IV breast cancer and HER2/neu positive lung cancer or ovarian cancer
3. Stage IV breast cancer manifested as local recurrence and/or distant metastases indicated failure of previous treatments for which curative or reliably effective palliative surgery, radiation therapy, or medical therapy was not available
4. Expected survival of at least 4 months
5. Previously received first-line chemotherapy (eg, anthracyclines, taxanes) whether or not previously treated on adjuvant chemotherapy
6. Previously received hormonal therapy if appropriate

Key exclusion criteria

1. Concurrent or recent chemotherapy (within 3 weeks), hormonal therapy, radiotherapy, immunotherapy, or general anesthesia/major surgery. Patients must have recovered from all known or expected toxicities from previous treatment and passed a treatment-free washout period of 3 weeks before starting this program (8 weeks for persons receiving nitrosourea or mitomycin)
2. History of anaphylactic reaction to any known or unknown antigen and clinical hypersensitivity to GM-CSF, interferon, yeast, beef, or to any components used in preparation of vaccine
3. Women who were pregnant or nursing
4. Concurrent second malignancy.

Period of Recruitment: 2005-2006

Table 4S. Endpoints, Sample Size and Reason for Termination.

Primary:

To assess clinical toxicity and feasibility of administration of this regimen with accrual of at least 9 evaluable patients.

Secondary:

To evaluate clinical responses, if any, after 3 vaccines and at the conclusion of study, i.e., after inoculation #6; as well as time to progression, and survival

To assess immune responses, if any, as measured by DTH skin tests, ELISA assays for antibody to tumor vaccine, and flow-activated cell sorter assay for vaccine antigen-reactive T-cells.

Choice of Sample Size:

The optimal two-stage design of Simon was used to determine sample size and early termination criteria related to vaccine activity (Simon 1989; Simon 2001). This design minimizes the number of patients treated with a treatment of possible low activity. Assuming a baseline level of response however defined (e.g. clinical, immunological, etc.) of no more than 5% and a response rate of interest of 25%, a false-positive rate (alpha error) of 0.10 and a false-negative rate of 0.10 (power of 0.90), this design calls for 9 patients in a first stage and a maximum of 24 patients. In the first stage, 9 assessable patients are entered and treated. If no responses are observed, the trial is terminated and the regimen is declared inactive. Otherwise, accrual continues to a total of 24 assessable patients. If the total number of clinical responses is at least 3, the regimen is considered clinically active. With this design, the probability of early termination based on activity/response is 0.63 when the true response level is no greater than 5%. This design, with a target response rate of 25% and baseline rate of 5%, is considered by Simon, et al. (2001) to be "reasonable for many initial vaccine trials"(p. 1850).

Simon R. Optimal two-stage designs for phase II clinical trials. *Control Clin Trials*, 1989, 10:1-10.

Simon RM, Steinberg SM, Hamilton M, Hildesheim A, Khleif S, Kwak LW, Mackall CL, Schlom J, Topalian SL, Berzofsky JA. Clinical trial designs for the early clinical development of therapeutic cancer vaccines. *J Clin Oncol* 2001; 19: 1848-1854.

Reason Study was Terminated: Medical Center ceased funding all research.

Table 5S. Baseline and Demographic Characteristics.

Parameters	A001	A002	A003	B001
Age, y	72	58	72	60
Gender	Female	Female	Female	Female
Ethnicity	Non-Latino	NA	NA	NA
Race	White	White	White	White
**Diagnosis at Baseline**				
Type of cancer	Breast cancer	Breast cancer	Ovarian cancer	Breast cancer
Stage as per AJCC	IV	IV	IV	IV
HER2/neu	2+/3	2+	2-3+/3	2-3+
Estrogen receptor	Negative	Positive	-	Positive
Progesterone receptor	Moderately positive	Focal Positive	-	Negative
Location of first metastasis	Chest wall	Breast nodes	Abdomen	Bone
Histology	Ductal	Ductal	-	Ductal
Lymphovascular invasion (Yes / No)	No	No	Yes	-
Tumor Grade (I, II, III)	III	II - Moderate	Not done	-
Metastatic status	Recurrent	Recurrent	Recurrent	Persistent
ECOG	0-1	0-1	1	0

NOTE: AJCC = American Joint Committee on Cancer, ECOG = Eastern Cooperative Oncology Group

SUPPLEMENTARY METHODS

We used GENEVESTIGATOR® (https://genevestigator.com**)** [1, 2] to identify breast cancer studies with grading information to identify signatures of genes expressed either ≥2 times *higher* or ≥2 times *lower* in in grade 3 tumors compared to grade 1 or 2 tumors, using a false-discovery rate (FDR) of 0.01. See **Supplementary Data Sheet 2** for detailed methods. Using these criteria with the NCBI GEO DataSets GSE1456 (86 grade 1 or 2, 61 grade 3 samples), GSE4922 (232 grade 1 or 2, 55 grade 3 samples), GSE7390 (113 grade 1 or 2, 83 grade 3 samples), GSE19615 (51 grade 1 or 2, 64 grade 3 samples), GSE20711 (18 grade 1 or 2, 70 grade 3 samples) and GSE31448 (133 grade 1 or 2, 124 grade 3 samples), and with the ArrayExpress data set E-MTAB-365 (318 grade 1 or 2, 199 grade 3 samples, 55 genes with probe sets unambiguously mapped to unique genes were present in the signatures of at least 5 of the 7 data sets. From these 55 genes, referred to as the CS55 "consensus signature" (**Data Sheet 2, Supplementary Table S2**), 54 genes (CS54 consensus signature) were represented in a quantile-normalized data set used previously (to generate Fig. **8** in [3]). From this quantile-normalized data set, for each of the 54 genes, the Illumina probe with the highest background-subtracted maximum value across all "representative samples" (defined in [3]) was selected. From this new dataset with 1 probe per gene, the *median* values of the 22 SV-BR-1-GM samples and the arithmetic *means* of the MDA-MB-231 (GSM1177035, GSM1177036, GSM1177037, GSM1177038, GSM1177039, GSM1177040), MDA-MB-468 (GSM1177044, GSM1177045¸ GSM1177046), and MCF-7 (GSM1177053, GSM1177054, GSM1177055, GSM1177056, GSM1177057, GSM1177058) and MCF-10A (GSM1177029, GSM1177030) samples (derived from the NCBI GEO Data Set GSE48398) were determined and used to calculate the Relative Molecular Grade (RMG). The RMG is defined as the sum (S) of the following values for each gene: The quantile-normalized expression level relative to the maximum expression value (max_{spls_gene}) across the 5 samples (SV-BR-1-GM, MDA-MB-231, MDA-MB-468, MCF-7, MCF-10A) (exp_{gene}), multiplied by the average fold-change ($avg(fc_{gene})$) across the 5-7 studies in the meta-analysis, relative to the

sum (S) of the average fold-changes for each set (fc_{set_1}: higher expressed in grade 1 or 2, positive fold-change values; fc_{set_2}: higher expressed in grade 3, negative fold-change values) (see formula below).

The resulting values were then adjusted to fit in a linear 0 (lowest "molecular grade") to 100 (highest "molecular grade") scale. For the calculation of the RMGs, background-subtracted normalized values were used (as defined elsewhere [3]), and only genes of the 54-gene signature were included for which the maximum expression values across the 5 samples was at least 1.5 times the background value.

$$RMG = S\{[(exp_{gene} / max_{spls\_gene}) * avg(fc_{gene})] / S(fc_{set\_1}, fc_{set\_2})\}, \text{ adjusted to fit in a 0 to 100 scale}$$

SUPPLEMENTARY TABLE S2: CS55 AND CS54 CONSENSUS SIGNATURES

Genes differentially expressed in grade 1 or 2 vs. grade 3 breast tumors, as determined using GENEVESTIGATOR® (Nebion; Schlieren, Switzerland). See text for details. The "Mean Log2-ratio" refers to the Log2 fold-changes in gene expression comparing Grade 1 or 2 with grade 3. HGNC, HUGO Gene Nomenclature Committee; *in CS55 but not in CS54 signature.

**Higher in Grade 1 or 2 than in Grade 3**	
**HGNC Gene Symbol**	**Mean Log2-ratio**
SCUBE2*	2.407
ESR1	2.339
NAT1	2.066
NPY1R	1.838
STC2	1.792
CA12	1.783
CPB1	1.757
PIP	1.746
DNAJC12	1.721
MAPT	1.606
AGR2	1.600
OGN	1.569
RNU4-46P	1.564
AGTR1	1.533
CXCL14	1.525
TBC1D9	1.503
ABAT	1.499
MLPH	1.451
ERBB4	1.433
LRRC17	1.410
GATA3	1.384
DACH1	1.372
PTGER3	1.367

**Higher in Grade 3 than in Grade 1 or 2**	
**HGNC Gene Symbol**	**Mean Log2-ratio**
MMP1	-1.981
S100A8	-1.962
CDC20	-1.577
TTK	-1.544
ASPM	-1.535
TOP2A	-1.533
RRM2	-1.513
DLGAP5	-1.472
TPX2	-1.453
MCM10	-1.448
FOXM1	-1.426
CENPA	-1.407
INAVA	-1.402
MELK	-1.395
CCNB2	-1.393
CEP55	-1.385
BIRC5	-1.372
ADAMDEC1	-1.355
BUB1	-1.348
SLC7A5	-1.341
UBE2C	-1.323
CXCL11	-1.322
AURKA	-1.311

CACNA1D	1.343
CX3CR1	1.331
NOVA1	1.211
PPP1R3C	1.175
KIF5C	1.171

CALML5	-1.304
PRC1	-1.295
CENPF	-1.282
CXCL10	-1.248

REFERENCES

[1] Hruz, T., et al., *Genevestigator v3: a reference expression database for the meta-analysis of transcriptomes.* Adv Bioinformatics, 2008. **2008**: p. 420747.

[2] Wang, S., et al., *Prediction of co-expression genes and integrative analysis of gene microarray and proteomics profile of Keshan disease.* Sci Rep, 2018. **8**(1): p. 231.

[3] Lacher, M.D., et al., *SV-BR-1-GM, a Clinically Effective GM-CSF-Secreting Breast Cancer Cell Line, Expresses an Immune Signature and Directly Activates CD4(+) T Lymphocytes.* Front Immunol, 2018. **9**: p. 776.
