# Supplementary material for: Regression of Breast Cancer Metastases Following Treatment with Irradiated SV-BR-1-GM, a GM-CSF Overexpressing Breast Cancer Cell Line: Intellectual Property and Immune Markers of Response
Source: Recent Pat Anticancer Drug Discov. 2023 Dec 28;18(2):224–40. doi: 10.2174/1574892817666220518123331 (PMC10009895; doi:10.2174/1574892817666220518123331)
Supplement: Supplementary file 1 [file PRA-18-224_SD1.zip › PRA-18-224_SD1/Wiseman_et_al_DataSheet_3_Final_PRA_post_review.pdf]

Literature Review References

Note that:

- For the phase II studies, Berntsen *et al.*, 2008 and Berntsen *et al.*, 2010 describe the same patients. Therefore, the indicated 148 references reflect only 147 studies.

- For the phase III studies, Wallack *et al.*, 1995 and Wallack *et al.*, 1998 describe the same patients, and Doehn *et al.*, 2003 and Jocham *et al.*, 2004 describe the same patients. Therefore, the indicated 18 references reflect only 16 studies.

Phase II Studies

1. Anguille S, Van de Velde AL, Smits EL, Van Tendeloo VF, Juliusson G, Cools N, Nijs G, Stein B, Lion E, Van Driessche A, Vandenbosch I, Verlinden A, Gadisseur AP, Schroyens WA, Muylle L, Vermeulen K, Maes MB, Deiteren K, Malfait R, Gostick E, Lammens M, Couttenye MM, Jorens P, Goossens H, Price DA, Ladell K, Oka Y, Fujiki F, Oji Y, Sugiyama H, Berneman ZN. Dendritic cell vaccination as postremission treatment to prevent or delay relapse in acute myeloid leukemia. Blood. 2017 Oct 12;130(15):1713-1721.
2. Antonarakis ES, Kibel AS, Yu EY, Karsh LI, Elfiky A, Shore ND, Vogelzang NJ, Corman JM, Millard FE, Maher JC, Chang NN, DeVries T, Sheikh NA, Drake CG; STAND Investigators. Sequencing of Sipuleucel-T and Androgen Deprivation Therapy in Men with Hormone-Sensitive Biochemically Recurrent Prostate Cancer: A Phase II Randomized Trial. Clin Cancer Res. 2017 May 15;23(10):2451-2459. doi: 10.1158/1078-0432.CCR-16-1780. Epub 2016 Nov 10. PubMed PMID: 27836866.
3. Ardon H, Van Gool SW, Verschuere T, Maes W, Fieuws S, Sciot R, Wilms G, Demaerel P, Goffin J, Van Calenbergh F, Menten J, Clement P, Debiec-Rychter M, De Vleeschouwer S. Integration of autologous dendritic cell-based immunotherapy in the standard of care treatment for patients with newly diagnosed glioblastoma: results of the HGG-2006 phase I/II trial. Cancer Immunol Immunother. 2012 Nov;61(11):2033-44. doi: 10.1007/s00262-012-1261-1. Epub 2012 Apr 22. PubMed PMID: 22527250.
4. Avigan DE, Vasir B, George DJ, Oh WK, Atkins MB, McDermott DF, Kantoff PW, Figlin RA, Vasconcelles MJ, Xu Y, Kufe D, Bukowski RM. Phase I/II study of vaccination with electrofused allogeneic dendritic cells/autologous tumor-derived cells in patients with stage IV renal cell carcinoma. J Immunother. 2007 Oct;30(7):749-61. PubMed PMID: 17893567.
5. Babatz J, Röllig C, Löbel B, Folprecht G, Haack M, Günther H, Köhne CH, Ehninger G, Schmitz M, Bornhäuser M. Induction of cellular immune responses against carcinoembryonic antigen in patients with metastatic tumors after vaccination with altered peptide ligand-loaded dendritic cells. Cancer Immunol Immunother. 2006 Mar;55(3):268-76. Epub 2005 Jul 21. PubMed PMID: 16034561.

6. Baek S, Kim CS, Kim SB, Kim YM, Kwon SW, Kim Y, Kim H, Lee H. Combination therapy of renal cell carcinoma or breast cancer patients with dendritic cell vaccine and IL-2: results from a phase I/II trial. J Transl Med. 2011 Oct 20;9:178. doi: 10.1186/1479-5876-9-178. PubMed PMID: 22013914; PubMed Central PMCID: PMC3213212.
7. Baek S, Kim YM, Kim SB, Kim CS, Kwon SW, Kim Y, Kim H, Lee H. Therapeutic DC vaccination with IL-2 as a consolidation therapy for ovarian cancer patients: a phase I/II trial. Cell Mol Immunol. 2015 Jan;12(1):87-95. doi: 10.1038/cmi.2014.40. Epub 2014 Jun 30. PubMed PMID: 24976269; PubMed Central PMCID: PMC4654372.
8. Baldueva IA, Novik AV, Moiseenko VM, Nekhaeva TL, Danilova AB, Danilov AO, Protsenko SA, Petrova TIu, Uleĭskaia GI, Shchekina LA, Semenova AI, Mikhaĭlichenko TD, Teletaeva GM, Zhabina AS, Volkov NV, Komarov IuI. [Phase II clinical trial of autologous dendritic cell vaccine with immunologic adjuvant in cutaneous melanoma patients]. Vopr Onkol. 2012;58(2):212-21. Russian. PubMed PMID: 22774527.
9. Bapsy PP, Sharan B, Kumar C, Das RP, Rangarajan B, Jain M, Suresh Attili VS, Subramanian S, Aggarwal S, Srivastava M, Vaid A. Open-label, multi-center, non-randomized, single-arm study to evaluate the safety and efficacy of dendritic cell immunotherapy in patients with refractory solid malignancies, on supportive care. Cytotherapy. 2014 Feb;16(2):234-44. doi: 10.1016/j.jcyt.2013.11.013. PubMed PMID: 24438902.
10. Barrou B, Benoît G, Ouldkaci M, Cussenot O, Salcedo M, Agrawal S, Massicard S, Bercovici N, Ericson ML, Thiounn N. Vaccination of prostatectomized prostate cancer patients in biochemical relapse, with autologous dendritic cells pulsed with recombinant human PSA. Cancer Immunol Immunother. 2004 May;53(5):453-60. Epub 2004 Feb 4. PubMed PMID: 14760510.
11. Bercovici N, Haicheur N, Massicard S, Vernel-Pauillac F, Adotevi O, Landais D, Gorin I, Robert C, Prince HM, Grob JJ, Leccia MT, Lesimple T, Wijdenes J, Bartholeyns J, Fridman WH, Salcedo M, Ferries E, Tartour E. Analysis and characterization of antitumor T-cell response after administration of dendritic cells loaded with allogeneic tumor lysate to metastatic melanoma patients. J Immunother. 2008 Jan;31(1):101-12. PubMed PMID: 18157017.
12. Berntsen A, Brimnes MK, thor Straten P, Svane IM. Increase of circulating CD4+CD25highFoxp3+ regulatory T cells in patients with metastatic renal cell carcinoma during treatment with dendritic cell vaccination and low-dose interleukin-2. J Immunother. 2010 May;33(4):425-34. doi: 10.1097/CJI.0b013e3181cd870f. PubMed PMID: 20386464.
13. Berntsen A, Trepiakas R, Wenandy L, Geertsen PF, thor Straten P, Andersen MH, Pedersen AE, Claesson MH, Lorentzen T, Johansen JS, Svane IM. Therapeutic dendritic cell vaccination of patients with metastatic renal cell carcinoma: a clinical phase 1/2 trial. J Immunother. 2008 Oct;31(8):771-80. doi: 10.1097/CJI.0b013e3181833818. PubMed PMID: 18779742.
14. Bjoern J, Brimnes MK, Andersen MH, Thor Straten P, Svane IM. Changes in peripheral blood level of regulatory T cells in patients with malignant melanoma during treatment with dendritic cell vaccination and low-dose IL-2. Scand J Immunol. 2011 Mar;73(3):222-33. doi: 10.1111/j.1365-3083.2010.02494.x. PubMed PMID: 21204893.

15. Bol KF, van den Bosch T, Schreibelt G, Mensink HW, Keunen JE, Kiliç E, Japing WJ, Geul KW, Westdorp H, Boudewijns S, Croockewit SA, van Rossum MM, de Goede AL, Naus NC, van der Graaf WT, Gerritsen WR, de Klein A, Punt CJ, Figdor CG, Cohen VM, Paridaens D, de Vries IJ. Adjuvant Dendritic Cell Vaccination in High-Risk Uveal Melanoma. Ophthalmology. 2016 Oct;123(10):2265-7. doi: 10.1016/j.ophtha.2016.06.027. Epub 2016 Jul 29. PubMed PMID: 27476772.
16. Boudewijns S, Bloemendal M, de Haas N, Westdorp H, Bol KF, Schreibelt G, Aarntzen EHJG, Lesterhuis WJ, Gorris MAJ, Croockewit A, van der Woude LL, van Rossum MM, Welzen M, de Goede A, Hato SV, van der Graaf WTA, Punt CJA, Koornstra RHT, Gerritsen WR, Figdor CG, de Vries IJM. Autologous monocyte-derived DC vaccination combined with cisplatin in stage III and IV melanoma patients: a prospective, randomized phase 2 trial. Cancer Immunol Immunother. 2020 Mar;69(3):477-488. doi: 10.1007/s00262-019-02466-x. Epub 2020 Jan 24. PubMed PMID: 31980913; PubMed Central PMCID: PMC7044256.
17. Brill TH, Kübler HR, Pohla H, Buchner A, Fend F, Schuster T, van Randenborgh H, Paul R, Kummer T, Plank C, Eisele B, Breul J, Hartung R, Schendel DJ, Gansbacher B. Therapeutic vaccination with an interleukin-2-interferon-gamma-secreting allogeneic tumor vaccine in patients with progressive castration-resistant prostate cancer: a phase I/II trial. Hum Gene Ther. 2009 Dec;20(12):1641-51. doi: 10.1089/hum.2009.101. PubMed PMID: 19671000.
18. Buchroithner J, Erhart F, Pichler J, Widhalm G, Preusser M, Stockhammer G et al (2018) Audencel Immunotherapy Based on Dendritic Cells Has No Effect on Overall and Progression-Free Survival in Newly Diagnosed Glioblastoma: A Phase II Randomized Trial. Cancers 10:372 Multidisciplinary Digital Publishing Institute; 2018 [cited 2018 Oct 8];10:372. Available from: https://www.mdpi.com/2072-6694/10/10/372
19. Burgdorf SK. Dendritic cell vaccination of patients with metastatic colorectal cancer. Dan Med Bull. 2010 Sep;57(9):B4171. PubMed PMID: 20816019.
20. Caballero-Baños M, Benitez-Ribas D, Tabera J, Varea S, Vilana R, Bianchi L, Ayuso JR, Pagés M, Carrera G, Cuatrecasas M, Martin-Richard M, Cid J, Lozano M, Castells A, García-Albéniz X, Maurel J, Vilella R. Phase II randomised trial of autologous tumour lysate dendritic cell plus best supportive care compared with best supportive care in pre-treated advanced colorectal cancer patients. Eur J Cancer. 2016 Sep;64:167-74.
21. Chang AE, Li Q, Jiang G, Sayre DM, Braun TM, Redman BG. Phase II trial of autologous tumor vaccination, anti-CD3-activated vaccine-primed lymphocytes, and interleukin-2 in stage IV renal cell cancer. J Clin Oncol. 2003 Mar 1;21(5):884-90. PubMed PMID: 12610189.
22. Chang CN, Huang YC, Yang DM, Kikuta K, Wei KJ, Kubota T, Yang WK. A phase I/II clinical trial investigating the adverse and therapeutic effects of a postoperative autologous dendritic cell tumor vaccine in patients with malignant glioma. J Clin Neurosci. 2011 Aug;18(8):1048-54. doi: 10.1016/j.jocn.2010.11.034. Epub 2011 Jun 28. PubMed PMID: 21715171.
23. Charles J, Chaperot L, Hannani D, Bruder Costa J, Templier I, Trabelsi S, Gil H, Moisan A, Persoons V, Hegelhofer H, Schir E, Quesada JL, Mendoza C, Aspord C, Manches O,

Coulie PG, Khammari A, Dreno B, Leccia MT, Plumas J. An innovative plasmacytoid dendritic cell line-based cancer vaccine primes and expands antitumor T-cells in melanoma patients in a first-in-human trial. Oncoimmunology. 2020 Apr 12;9(1):1738812. doi: 10.1080/2162402X.2020.1738812. eCollection 2020.

24. Chia WK, Wang WW, Teo M, Tai WM, Lim WT, Tan EH, Leong SS, Sun L, Chen JJ, Gottschalk S, Toh HC. A phase II study evaluating the safety and efficacy of an adenovirus-ΔLMP1-LMP2 transduced dendritic cell vaccine in patients with advanced metastatic nasopharyngeal carcinoma. Ann Oncol. 2012 Apr;23(4):997-1005. doi: 10.1093/annonc/mdr341. Epub 2011 Aug 4. PubMed PMID: 21821548; PubMed Central PMCID: PMC3314324.
25. Chiappori AA, Williams CC, Gray JE, Tanvetyanon T, Haura EB, Creelan BC, Thapa R, Chen DT, Simon GR, Bepler G, Gabrilovich DI, Antonia SJ. Randomized-controlled phase II trial of salvage chemotherapy after immunization with a TP53-transfected dendritic cell-based vaccine (Ad.p53-DC) in patients with recurrent small cell lung cancer. Cancer Immunol Immunother. 2019 Mar;68(3):517-527.
26. Cho DY, Yang WK, Lee HC, Hsu DM, Lin HL, Lin SZ, Chen CC, Harn HJ, Liu CL, Lee WY, Ho LH. Adjuvant immunotherapy with whole-cell lysate dendritic cells vaccine for glioblastoma multiforme: a phase II clinical trial. World Neurosurg. 2012 May-Jun;77(5-6):736-44. doi: 10.1016/j.wneu.2011.08.020. Epub 2011 Nov 7. PubMed PMID: 22120301.
27. Chodon T, Comin-Anduix B, Chmielowski B, Koya RC, Wu Z, Auerbach M, Ng C, Avramis E, Seja E, Villanueva A, McCannel TA, Ishiyama A, Czernin J, Radu CG, Wang X, Gjertson DW, Cochran AJ, Cornetta K, Wong DJ, Kaplan-Lefko P, Hamid O, Samlowski W, Cohen PA, Daniels GA, Mukherji B, Yang L, Zack JA, Kohn DB, Heath JR, Glaspy JA, Witte ON, Baltimore D, Economou JS, Ribas A. Adoptive transfer of MART-1 T-cell receptor transgenic lymphocytes and dendritic cell vaccination in patients with metastatic melanoma. Clin Cancer Res. 2014 May 1;20(9):2457-65. doi: 10.1158/1078-0432.CCR-13-3017. Epub 2014 Mar 14. PubMed PMID: 24634374; PubMed Central PMCID: PMC4070853.
28. Chu CS, Boyer J, Schullery DS, Gimotty PA, Gamerman V, Bender J, Levine BL, Coukos G, Rubin SC, Morgan MA, Vonderheide RH, June CH. Phase I/II randomized trial of dendritic cell vaccination with or without cyclophosphamide for consolidation therapy of advanced ovarian cancer in first or second remission. Cancer Immunol Immunother. 2012 May;61(5):629-41. doi: 10.1007/s00262-011-1081-8. Epub 2011 Oct 22. PubMed PMID: 22021066.
29. Coosemans A, Vanderstraeten A, Tuyaerts S, Verschuere T, Moerman P, Berneman ZN, Vergote I, Amant F, VAN Gool SW. Wilms' Tumor Gene 1 (WT1)--loaded dendritic cell immunotherapy in patients with uterine tumors: a phase I/II clinical trial. Anticancer Res. 2013 Dec;33(12):5495-500. PubMed PMID: 24324087.
30. Creelan BC, Antonia S, Noyes D, Hunter TB, Simon GR, Bepler G, Williams CC, Tanvetyanon T, Haura EB, Schell MJ, Chiappori A. Phase II trial of a GM-CSF-producing and CD40L-expressing bystander cell line combined with an allogeneic tumor cell-based vaccine for refractory lung adenocarcinoma. J Immunother. 2013 Oct;36(8):442-50. doi:

10.1097/CJI.0b013e3182a80237. PubMed PMID: 23994887; PubMed Central PMCID: PMC3846277.
31. Curti A, Tosi P, Comoli P, Terragna C, Ferri E, Cellini C, Massaia M, D'Addio A, Giudice V, Di Bello C, Cavo M, Conte R, Gugliotta G, Baccarani M, Lemoli RM. Phase I/II clinical trial of sequential subcutaneous and intravenous delivery of dendritic cell vaccination for refractory multiple myeloma using patient-specific tumour idiotype protein or idiotype (VDJ)-derived class I-restricted peptides. Br J Haematol. 2007 Nov;139(3):415-24. PubMed PMID: 17910631.
32. Dillman R, Barth N, Selvan S, Beutel L, de Leon C, DePriest C, Peterson C, Nayak S. Phase I/II trial of autologous tumor cell line-derived vaccines for recurrent or metastatic sarcomas. Cancer Biother Radiopharm. 2004 Oct;19(5):581-8. PubMed PMID: 15650450.
33. Dillman R, Selvan S, Schiltz P, Peterson C, Allen K, Depriest C, McClay E, Barth N, Sheehy P, de Leon C, Beutel L. Phase I/II trial of melanoma patient-specific vaccine of proliferating autologous tumor cells, dendritic cells, and GM-CSF: planned interim analysis. Cancer Biother Radiopharm. 2004 Oct;19(5):658-65. PubMed PMID: 15650459.
34. Dillman RO, Barth NM, VanderMolen LA, Garfield DH, De Leon C, O'Connor AA, Mahdavi K, Nayak SK. Treatment of kidney cancer with autologous tumor cell vaccines of short-term cell lines derived from renal cell carcinoma. Cancer Biother Radiopharm. 2001 Feb;16(1):47-54. PubMed PMID: 11279797.
35. Dillman RO, Beutel LD, Barth NM, de Leon C, O'Connor AA, DePriest C, Nayak SK. Irradiated cells from autologous tumor cell lines as patient-specific vaccine therapy in 125 patients with metastatic cancer: induction of delayed-type hypersensitivity to autologous tumor is associated with improved survival. Cancer Biother Radiopharm. 2002 Feb;17(1):51-66. PubMed PMID: 11915174.
36. Dillman RO, Cornforth AN, Nistor GI, McClay EF, Amatruda TT, Depriest C. Randomized phase II trial of autologous dendritic cell vaccines versus autologous tumor cell vaccines in metastatic melanoma: 5-year follow up and additional analyses. J Immunother Cancer. 2018 Mar 6;6(1):19. doi: 10.1186/s40425-018-0330-1. PubMed PMID: 29510745; PubMed Central PMCID: PMC5840808.
37. Dillman RO, DeLeon C, Beutel LD, Barth NM, Schwartzberg LS, Spitler LE, Garfield DH, O'Connor AA, Nayak SK. Short-term autologous tumor cell lines for the active specific immunotherapy of patients with metastatic melanoma. Crit Rev Oncol Hematol. 2001 Jul-Aug;39(1-2):115-23. PubMed PMID: 11418308.
38. Dillman RO, McClay EF, Barth NM, Amatruda TT, Schwartzberg LS, Mahdavi K, de Leon C, Ellis RE, DePriest C. Dendritic Versus Tumor Cell Presentation of Autologous Tumor Antigens for Active Specific Immunotherapy in Metastatic Melanoma: Impact on Long-Term Survival by Extent of Disease at the Time of Treatment. Cancer Biother Radiopharm. 2015 Jun;30(5):187-94. doi: 10.1089/cbr.2015.1843. PubMed PMID: 26083950; PubMed Central PMCID: PMC4492594.
39. Dillman RO, Selvan SR, Schiltz PM, McClay EF, Barth NM, DePriest C, de Leon C, Mayorga C, Cornforth AN, Allen K. Phase II trial of dendritic cells loaded with antigens from self-renewing, proliferating autologous tumor cells as patient-specific antitumor

vaccines in patients with metastatic melanoma: final report. Cancer Biother Radiopharm. 2009 Jun;24(3):311-9. doi: 10.1089/cbr.2008.0599.

40. Dillman RO, Wiemann M, Nayak SK, DeLeon C, Hood K, DePriest C. Interferon-gamma or granulocyte-macrophage colony-stimulating factor administered as adjuvants with a vaccine of irradiated autologous tumor cells from short-term cell line cultures: a randomized phase 2 trial of the cancer biotherapy research group. J Immunother. 2003 Jul-Aug;26(4):367-73. PubMed PMID: 12843799.
41. Dudek AZ, Mescher MF, Okazaki I, Math VT, Luo X, Curtsinger JM, Miller JS. Autologous large multivalent immunogen vaccine in patients with metastatic melanoma and renal cell carcinoma. Am J Clin Oncol. 2008 Apr;31(2):173-81. doi: 10.1097/COC.0b013e3181573e6b. PubMed PMID: 18391603.
42. Eaton JD, Perry MJ, Nicholson S, Guckian M, Russell N, Whelan M, Kirby RS. Allogeneic whole-cell vaccine: a phase I/II study in men with hormone-refractory prostate cancer. BJU Int. 2002 Jan;89(1):19-26. PubMed PMID: 11849155.
43. Elias EG, Zapas JL, McCarron EC, Beam SL, Hasskamp JH, Culpepper WJ. Sequential administration of GM-CSF (Sargramostim) and IL-2 +/- autologous vaccine as adjuvant therapy in cutaneous melanoma: an interim report of a phase II clinical trial. Cancer Biother Radiopharm. 2008 Jun;23(3):285-91. doi: 10.1089/cbr.2007.0438. PubMed PMID: 18593361.
44. Ellebaek E, Engell-Noerregaard L, Iversen TZ, Froesig TM, Munir S, Hadrup SR, Andersen MH, Svane IM. Metastatic melanoma patients treated with dendritic cell vaccination, Interleukin-2 and metronomic cyclophosphamide: results from a phase II trial. Cancer Immunol Immunother. 2012 Oct;61(10):1791-804. Epub 2012 Mar 20. PubMed PMID: 22426890.
45. Elliott GT, McLeod RA, Perez J, Von Eschen KB. Interim results of a phase II multicenter clinical trial evaluating the activity of a therapeutic allogeneic melanoma vaccine (theraccine) in the treatment of disseminated malignant melanoma. Semin Surg Oncol. 1993 May-Jun;9(3):264-72. PubMed PMID: 8516615. → Abstract only available
46. Engelstein R, Merims S, Eisenberg G, Cohen J, Frank S, Hamburger T, Frankenburg S, Ron I, Isacson R, Grenader T, Steinberg H, Cohen CJ, Peretz T, Lotem M. Immune Monitoring of Patients Treated With a Whole-Cell Melanoma Vaccine Engineered to Express 4-1BBL. J Immunother. 2016 Oct;39(8):321-8.
47. Fishman M, Hunter TB, Soliman H, Thompson P, Dunn M, Smilee R, Farmelo MJ, Noyes DR, Mahany JJ, Lee JH, Cantor A, Messina J, Seigne J, Pow-Sang J, Janssen W, Antonia SJ. Phase II trial of B7-1 (CD-86) transduced, cultured autologous tumor cell vaccine plus subcutaneous interleukin-2 for treatment of stage IV renal cell carcinoma. J Immunother. 2008 Jan;31(1):72-80. PubMed PMID: 18157014.
48. Flörcken A, Kopp J, van Lessen A, Movassaghi K, Takvorian A, Jöhrens K, Möbs M, Schönemann C, Sawitzki B, Egerer K, Dörken B, Pezzutto A, Westermann J. Allogeneic partially HLA-matched dendritic cells pulsed with autologous tumor cell lysate as a vaccine in metastatic renal cell cancer: a clinical phase I/II study. Hum Vaccin Immunother. 2013 Jun;9(6):1217-27. doi: 10.4161/hv.24149. Epub 2013 Mar 4. PubMed PMID: 23458999; PubMed Central PMCID: PMC3901809.

49. Fong L, Carroll P, Weinberg V, Chan S, Lewis J, Corman J, Amling CL, Stephenson RA, Simko J, Sheikh NA, Sims RB, Frohlich MW, Small EJ. Activated lymphocyte recruitment into the tumor microenvironment following preoperative sipuleucel-T for localized prostate cancer. J Natl Cancer Inst. 2014 Sep 24;106(11). pii: dju268. doi: 10.1093/jnci/dju268. Print 2014 Nov. Erratum in: J Natl Cancer Inst. 2014 Nov;106(11):dju372 doi:10.1093/jnci/dju372. PubMed PMID: 25255802; PubMed Central PMCID: PMC4241888.
50. Fukuda K, Funakoshi T, Sakurai T, Nakamura Y, Mori M, Tanese K, Tanikawa A, Taguchi J, Fujita T, Okamoto M, Amagai M, Kawakami Y. Peptide-pulsed dendritic cell vaccine in combination with carboplatin and paclitaxel chemotherapy for stage IV melanoma. Melanoma Res. 2017 Aug;27(4):326-334.
51. Geskin LJ, Damiano JJ, Patrone CC, Butterfield LH, Kirkwood JM, Falo LD. Three antigen-loading methods in dendritic cell vaccines for metastatic melanoma. Melanoma Res. 2018 Jun;28(3):211-221.
52. Greene JM, Schneble EJ, Jackson DO, Hale DF, Vreeland TJ, Flores M, Martin J, Herbert GS, Hardin MO, Yu X, Wagner TE, Peoples GE. A phase I/IIa clinical trial in stage IV melanoma of an autologous tumor-dendritic cell fusion (dendritoma) vaccine with low dose interleukin-2. Cancer Immunol Immunother. 2016 Apr;65(4):383-92.
53. Haenssle HA, Krause SW, Emmert S, Zutt M, Kretschmer L, Schmidberger H, Andreesen R, Soruri A. Hybrid cell vaccination in metastatic melanoma: clinical and immunologic results of a phase I/II study. J Immunother. 2004 Mar-Apr;27(2):147-55. PubMed PMID: 14770086.
54. Hanna MG Jr, Hoover HC Jr, Vermorken JB, Harris JE, Pinedo HM. Adjuvant active specific immunotherapy of stage II and stage III colon cancer with an autologous tumor cell vaccine: first randomized phase III trials show promise. Vaccine. 2001 Mar 21;19(17-19):2576-82. PubMed PMID: 11257395.
55. Hardacre JM, Mulcahy M, Small W, Talamonti M, Obel J, Krishnamurthi S, Rocha-Lima CS, Safran H, Lenz HJ, Chiorean EG. Addition of algenpantucel-L immunotherapy to standard adjuvant therapy for pancreatic cancer: a phase 2 study. J Gastrointest Surg. 2013 Jan;17(1):94-100; discussion p. 100-1. doi: 10.1007/s11605-012-2064-6. Epub 2012 Nov 15. PubMed PMID: 23229886.
56. Harris JE, Ryan L, Hoover HC Jr, Stuart RK, Oken MM, Benson AB 3rd, Mansour E, Haller DG, Manola J, Hanna MG Jr. Adjuvant active specific immunotherapy for stage II and III colon cancer with an autologous tumor cell vaccine: Eastern Cooperative Oncology Group Study E5283. J Clin Oncol. 2000 Jan;18(1):148-57. PubMed PMID: 10623705.
57. Hersey P, Halliday GM, Farrelly ML, DeSilva C, Lett M, Menzies SW. Phase I/II study of treatment with matured dendritic cells with or without low dose IL-2 in patients with disseminated melanoma. Cancer Immunol Immunother. 2008 Jul;57(7):1039-51. PubMed PMID: 18157724.
58. Hersey P, Menzies SW, Halliday GM, Nguyen T, Farrelly ML, DeSilva C, Lett M. Phase I/II study of treatment with dendritic cell vaccines in patients with disseminated melanoma. Cancer Immunol Immunother. 2004 Feb;53(2):125-34. Epub 2003 Nov 5. PubMed PMID: 14600790.

59. Higano CS, Corman JM, Smith DC, Centeno AS, Steidle CP, Gittleman M, Simons JW, Sacks N, Aimi J, Small EJ. Phase 1/2 dose-escalation study of a GM-CSF-secreting, allogeneic, cellular immunotherapy for metastatic hormone-refractory prostate cancer. Cancer. 2008 Sep 1;113(5):975-84. doi: 10.1002/cncr.23669. PubMed PMID: 18646045.
60. Höltl L, Ramoner R, Zelle-Rieser C, Gander H, Putz T, Papesh C, Nussbaumer W, Falkensammer C, Bartsch G, Thurnher M. Allogeneic dendritic cell vaccination against metastatic renal cell carcinoma with or without cyclophosphamide. Cancer Immunol Immunother. 2005 Jul;54(7):663-70. Epub 2004 Dec 17. PubMed PMID: 15918076.
61. Jacobs JF, Punt CJ, Lesterhuis WJ, Sutmuller RP, Brouwer HM, Scharenborg NM, Klasen IS, Hilbrands LB, Figdor CG, de Vries IJ, Adema GJ. Dendritic cell vaccination in combination with anti-CD25 monoclonal antibody treatment: a phase I/II study in metastatic melanoma patients. Clin Cancer Res. 2010 Oct 15;16(20):5067-78. doi: 10.1158/1078-0432.CCR-10-1757. Epub 2010 Aug 24. PubMed PMID: 20736326.
62. Jha G, Miller JS, Curtsinger JM, Zhang Y, Mescher MF, Dudek AZ. Randomized phase II study of IL-2 with or without an allogeneic large multivalent immunogen vaccine for the treatment of stage IV melanoma. Am J Clin Oncol. 2014 Jun;37(3):261-5. doi: 10.1097/COC.0b013e318277d5c8. PubMed PMID: 23241505; PubMed Central PMCID: PMC5547569.
63. Jiang XP, Yang DC, Elliott RL, Head JF. Vaccination with a mixed vaccine of autogenous and allogeneic breast cancer cells and tumor associated antigens CA15-3, CEA and CA125--results in immune and clinical responses in breast cancer patients. Cancer Biother Radiopharm. 2000 Oct;15(5):495-505. PubMed PMID: 11155821.
64. Katano M, Morisaki T, Koga K, Nakamura M, Onishi H, Matsumoto K, Tasaki A, Nakashima H, Akiyoshi T, Nakamura M. Combination therapy with tumor cell-pulsed dendritic cells and activated lymphocytes for patients with disseminated carcinomas. Anticancer Res. 2005 Nov-Dec;25(6A):3771-6. PubMed PMID: 16302738.
65. Khoury HJ, Collins RH Jr, Blum W, Stiff PS, Elias L, Lebkowski JS, Reddy A, Nishimoto KP, Sen D, Wirth ED 3rd, Case CC, DiPersio JF. Immune responses and long-term disease recurrence status after telomerase-based dendritic cell immunotherapy in patients with acute myeloid leukemia. Cancer. 2017 Aug 15;123(16):3061-3072. doi: 10.1002/cncr.30696. Epub 2017 Apr 14. PubMed PMID: 28411378.
66. Kitawaki T, Kadowaki N, Fukunaga K, Kasai Y, Maekawa T, Ohmori K, Kondo T, Maekawa R, Takahara M, Nieda M, Kuzushima K, Ishikawa T, Uchiyama T. A phase I/IIa clinical trial of immunotherapy for elderly patients with acute myeloid leukaemia using dendritic cells co-pulsed with WT1 peptide and zoledronate. Br J Haematol. 2011 Jun;153(6):796-9. doi: 10.1111/j.1365-2141.2010.08490.x. Epub 2011 Apr 8. PubMed PMID: 21477159.
67. Kongsted P, Borch TH, Ellebaek E, Iversen TZ, Andersen R, Met Ö, Hansen M, Lindberg H, Sengeløv L, Svane IM. Dendritic cell vaccination in combination with docetaxel for patients with metastatic castration-resistant prostate cancer: A randomized phase II study. Cytotherapy. 2017 Apr;19(4):500-513.
68. Koster BD, Santegoets SJAM, Harting J, Baars A, van Ham SM, Scheper RJ, Hooijberg E, de Gruijl TD, van den Eertwegh AJM. Autologous tumor cell vaccination combined with

systemic CpG-B and IFN-α promotes immune activation and induces clinical responses in patients with metastatic renal cell carcinoma: a phase II trial. Cancer Immunol Immunother. 2019 Jun;68(6):1025-1035.

69. Kuang M, Peng BG, Lu MD, Liang LJ, Huang JF, He Q, Hua YP, Totsuka S, Liu SQ, Leong KW, Ohno T. Phase II randomized trial of autologous formalin-fixed tumor vaccine for postsurgical recurrence of hepatocellular carcinoma. Clin Cancer Res. 2004 Mar 1;10(5):1574-9. PubMed PMID: 15014006.

70. Kuwabara K, Nishishita T, Morishita M, Oyaizu N, Yamashita S, Kanematsu T, Obara T, Mimura Y, Inoue Y, Kaminishi M, Kaga K, Amino N, Kitaoka M, Ito K, Miyauchi A, Noguchi S, Uchimaru K, Akagawa E, Watanabe N, Takahashi TA, Sato K, Inazawa T, Nakaoka T, Yamashita N. Results of a phase I clinical study using dendritic cell vaccinations for thyroid cancer. Thyroid. 2007 Jan;17(1):53-8. PubMed PMID: 17274750.

71. Kyte JA, Mu L, Aamdal S, Kvalheim G, Dueland S, Hauser M, Gullestad HP, Ryder T, Lislerud K, Hammerstad H, Gaudernack G. Phase I/II trial of melanoma therapy with dendritic cells transfected with autologous tumor-mRNA. Cancer Gene Ther. 2006 Oct;13(10):905-18. Epub 2006 May 5. PubMed PMID: 16710345.

72. Laurell A, Lönnemark M, Brekkan E, Magnusson A, Tolf A, Wallgren AC, Andersson B, Adamson L, Kiessling R, Karlsson-Parra A. Intratumorally injected pro-inflammatory allogeneic dendritic cells as immune enhancers: a first-in-human study in unfavourable risk patients with metastatic renal cell carcinoma. J Immunother Cancer. 2017 Jun 20;5:52. doi: 10.1186/s40425-017-0255-0. eCollection 2017. PubMed PMID: 28642820; PubMed Central PMCID: PMC5477104.

73. Le DT, Wang-Gillam A, Picozzi V, Greten TF, Crocenzi T, Springett G, Morse M, Zeh H, Cohen D, Fine RL, Onners B, Uram JN, Laheru DA, Lutz ER, Solt S, Murphy AL, Skoble J, Lemmens E, Grous J, Dubensky T Jr, Brockstedt DG, Jaffee EM. Safety and survival with GVAX pancreas prime and Listeria Monocytogenes-expressing mesothelin (CRS-207) boost vaccines for metastatic pancreatic cancer. J Clin Oncol. 2015 Apr 20;33(12):1325-33.

74. Lee JH, Lee Y, Lee M, Heo MK, Song JS, Kim KH, Lee H, Yi NJ, Lee KW, Suh KS, Bae YS, Kim YJ. A phase I/IIa study of adjuvant immunotherapy with tumour antigen-pulsed dendritic cells in patients with hepatocellular carcinoma. Br J Cancer. 2015 Dec 22;113(12):1666-76. doi: 10.1038/bjc.2015.430. Epub 2015 Dec 10. PubMed PMID: 26657650; PubMed Central PMCID: PMC4702003.

75. Lemoine FM, Cherai M, Giverne C, Dimitri D, Rosenzwajg M, Trebeden-Negre H, Chaput N, Barrou B, Thioun N, Gattegnio B, Selles F, Six A, Azar N, Lotz JP, Buzyn A, Sibony M, Delcourt A, Boyer O, Herson S, Klatzmann D, Lacave R. Massive expansion of regulatory T-cells following interleukin 2 treatment during a phase I-II dendritic cell-based immunotherapy of metastatic renal cancer. Int J Oncol. 2009 Sep;35(3):569-81. PubMed PMID: 19639177.

76. Lesimple T, Neidhard EM, Vignard V, Lefeuvre C, Adamski H, Labarrière N, Carsin A, Monnier D, Collet B, Clapisson G, Birebent B, Philip I, Toujas L, Chokri M, Quillien V. Immunologic and clinical effects of injecting mature peptide-loaded dendritic cells by

intralymphatic and intranodal routes in metastatic melanoma patients. Clin Cancer Res. 2006 Dec 15;12(24):7380-8. PubMed PMID: 17189411.

77. López MN, Pereda C, Segal G, Muñoz L, Aguilera R, González FE, Escobar A, Ginesta A, Reyes D, González R, Mendoza-Naranjo A, Larrondo M, Compán A, Ferrada C, Salazar-Onfray F. Prolonged survival of dendritic cell-vaccinated melanoma patients correlates with tumor-specific delayed type IV hypersensitivity response and reduction of tumor growth factor beta-expressing T cells. J Clin Oncol. 2009 Feb 20;27(6):945-52. doi: 10.1200/JCO.2008.18.0794. Epub 2009 Jan 12. PubMed PMID: 19139436.
78. Lotem M, Merims S, Frank S, Hamburger T, Nissan A, Kadouri L, Cohen J, Straussman R, Eisenberg G, Frankenburg S, Carmon E, Alaiyan B, Shneibaum S, Ozge Ayyildiz Z, Isbilen M, Mert Senses K, Ron I, Steinberg H, Smith Y, Shiloni E, Gure AO, Peretz T. Adjuvant Autologous Melanoma Vaccine for Macroscopic Stage III Disease: Survival, Biomarkers, and Improved Response to CTLA-4 Blockade. J Immunol Res. 2016;2016:8121985.
79. Luiten RM, Kueter EW, Mooi W, Gallee MP, Rankin EM, Gerritsen WR, Clift SM, Nooijen WJ, Weder P, van de Kasteele WF, Sein J, van den Berk PC, Nieweg OE, Berns AM, Spits H, de Gast GC. Immunogenicity, including vitiligo, and feasibility of vaccination with autologous GM-CSF-transduced tumor cells in metastatic melanoma patients. J Clin Oncol. 2005 Dec 10;23(35):8978-91. Epub 2005 Oct 31. PubMed PMID: 16260696.
80. Lutz E, Yeo CJ, Lillemoe KD, Biedrzycki B, Kobrin B, Herman J, Sugar E, Piantadosi S, Cameron JL, Solt S, Onners B, Tartakovsky I, Choi M, Sharma R, Illei PB, Hruban RH, Abrams RA, Le D, Jaffee E, Laheru D. A lethally irradiated allogeneic granulocyte-macrophage colony stimulating factor-secreting tumor vaccine for pancreatic adenocarcinoma. A Phase II trial of safety, efficacy, and immune activation. Ann Surg. 2011 Feb;253(2):328-35. doi: 10.1097/SLA.0b013e3181fd271c. PubMed PMID: 21217520; PubMed Central PMCID: PMC3085934.
81. Mackiewicz A, Mackiewicz J, Karczewska-Dzionk A, Laciak M, Kapcinska M, Wiznerowicz M, Burzykowski T, Zakowska M, Rose-John S, Mackiewicz A. Whole Cell Therapeutic Vaccine Modified With Hyper-IL6 for Combinational Treatment of Nonresected Advanced Melanoma. Medicine (Baltimore). 2015 May;94(21):e853.
82. Mackiewicz A, Mackiewicz J, Wysocki PJ, Wiznerowicz M, Kapcinska M, Laciak M, Rose-John S, Izycki D, Burzykowski T, Karczewska-Dzionk A. Long-term survival of high-risk melanoma patients immunized with a Hyper-IL-6-modified allogeneic whole-cell vaccine after complete resection. Expert Opin Investig Drugs. 2012 Jun;21(6):773-83. doi: 10.1517/13543784.2012.684753. Erratum in: Expert Opin Investig Drugs. 2012 Jul;21(7):1065. PubMed PMID: 22577889.
83. Mackiewicz J, Burzykowski T, Iżycki D, Mackiewicz A. Re-induction using whole cell melanoma vaccine genetically modified to melanoma stem cells-like beyond recurrence extends long term survival of high risk resected patients - updated results. J Immunother Cancer. 2018 Nov 29;6(1):134. → long-term follow-up of
84. Märten A, Flieger D, Renoth S, Weineck S, Albers P, Compes M, Schöttker B, Ziske C, Engelhart S, Hanfland P, Krizek L, Faber C, von Ruecker A, Müller S, Sauerbruch T,

Schmidt-Wolf IG. Therapeutic vaccination against metastatic renal cell carcinoma by autologous dendritic cells: preclinical results and outcome of a first clinical phase I/II trial. Cancer Immunol Immunother. 2002 Dec;51(11-12):637-44. Epub 2002 Oct 3. PubMed PMID: 12439609.

85. Märten A, Renoth S, Heinicke T, Albers P, Pauli A, Mey U, Caspari R, Flieger D, Hanfland P, Von Ruecker A, Eis-Hübinger AM, Müller S, Schwaner I, Lohmann U, Heylmann G, Sauerbruch T, Schmidt-Wolf IG. Allogeneic dendritic cells fused with tumor cells: preclinical results and outcome of a clinical phase I/II trial in patients with metastatic renal cell carcinoma. Hum Gene Ther. 2003 Mar 20;14(5):483-94. PubMed PMID: 12691613.

86. Meijer SL, Dols A, Urba WJ, Hu HM, Smith II JW, Vetto J, Wood W, Doran T, Chu Y, Sayaharuban P, Alvord WG, Fox BA. Adoptive cellular therapy with tumor vaccine draining lymph node lymphocytes after vaccination with HLA-B7/beta2-microglobulin gene-modified autologous tumor cells. J Immunother. 2002 Jul-Aug;25(4):359-72. PubMed PMID: 12142559.

87. Moiseyenko VM, Danilov AO, Baldueva IA, Danilova AB, Tyukavina NV, Larin SS, Kiselev SL, Orlova RV, Anisimov VV, Semenova AI, Shchekina LA, Gafton GI, Kochnev VA, Barchuk AS, Kanaev SV, Hanson KP, Georgiev GP. Phase I/II trial of gene therapy with autologous tumor cells modified with tag7/PGRP-S gene in patients with disseminated solid tumors: miscellaneous tumors. Ann Oncol. 2005 Jan;16(1):162-8. PubMed PMID: 15598955.

88. Mordoh J, Kairiyama C, Bover L, Solarolo E. Allogeneic cells vaccine increases disease-free survival in stage III melanoma patients. A non randomized phase II study. Medicina (B Aires). 1997;57(4):421-7. PubMed PMID: 9674264.

89. Morse MA, Niedzwiecki D, Marshall JL, Garrett C, Chang DZ, Aklilu M, Crocenzi TS, Cole DJ, Dessureault S, Hobeika AC, Osada T, Onaitis M, Clary BM, Hsu D, Devi GR, Bulusu A, Annechiarico RP, Chadaram V, Clay TM, Lyerly HK. A randomized phase II study of immunization with dendritic cells modified with poxvectors encoding CEA and MUC1 compared with the same poxvectors plus GM-CSF for resected metastatic colorectal cancer. Ann Surg. 2013 Dec;258(6):879-86. doi: 10.1097/SLA.0b013e318292919e. PubMed PMID: 23657083; PubMed Central PMCID: PMC3812363.

90. Motohashi S, Nagato K, Kunii N, Yamamoto H, Yamasaki K, Okita K, Hanaoka H, Shimizu N, Suzuki M, Yoshino I, Taniguchi M, Fujisawa T, Nakayama T. A phase I-II study of alpha-galactosylceramide-pulsed IL-2/GM-CSF-cultured peripheral blood mononuclear cells in patients with advanced and recurrent non-small cell lung cancer. J Immunol. 2009 Feb 15;182(4):2492-501. doi: 10.4049/jimmunol.0800126. PubMed PMID: 19201905.

91. Moviglia GA. Development of tumor B-cell lymphocyte hybridoma (TBH) autovaccination. Results of a phase I-II clinical trial. Transfus Sci. 1996 Dec;17(4):643-9. PubMed PMID: 10168565.

92. Muragaki Y, Maruyama T, Iseki H, Tanaka M, Shinohara C, Takakura K, Tsuboi K, Yamamoto T, Matsumura A, Matsutani M, Karasawa K, Shimada K, Yamaguchi N, Nakazato Y, Sato K, Uemae Y, Ohno T, Okada Y, Hori T. Phase I/IIa trial of autologous

formalin-fixed tumor vaccine concomitant with fractionated radiotherapy for newly diagnosed glioblastoma. Clinical article. J Neurosurg. 2011 Aug;115(2):248-55. doi: 10.3171/2011.4.JNS10377. Epub 2011 May 13. Erratum in: J Neurosurg. 2013 Mar;118(3):709. PubMed PMID: 21568657.

93. Murphy GP, Tjoa BA, Simmons SJ, Jarisch J, Bowes VA, Ragde H, Rogers M, Elgamal A, Kenny GM, Cobb OE, Ireton RC, Troychak MJ, Salgaller ML, Boynton AL. Infusion of dendritic cells pulsed with HLA-A2-specific prostate-specific membrane antigen peptides: a phase II prostate cancer vaccine trial involving patients with hormone-refractory metastatic disease. Prostate. 1999 Jan 1;38(1):73-8. PubMed PMID: 9973112.

94. Murphy GP, Tjoa BA, Simmons SJ, Ragde H, Rogers M, Elgamal A, Kenny GM, Troychak MJ, Salgaller ML, Boynton AL. Phase II prostate cancer vaccine trial: report of a study involving 37 patients with disease recurrence following primary treatment. Prostate. 1999 Apr 1;39(1):54-9. PubMed PMID: 10221267.

95. Narita M, Kanda T, Abe T, Uchiyama T, Iwafuchi M, Zheng Z, Liu A, Kaifu T, Kosugi S, Minagawa M, Itoh K, Takahashi M. Immune responses in patients with esophageal cancer treated with SART1 peptide-pulsed dendritic cell vaccine. Int J Oncol. 2015 Apr;46(4):1699-709.

96. Nemunaitis J, Jahan T, Ross H, Sterman D, Richards D, Fox B, Jablons D, Aimi J, Lin A, Hege K. Phase 1/2 trial of autologous tumor mixed with an allogeneic GVAX vaccine in advanced-stage non-small-cell lung cancer. Cancer Gene Ther. 2006 Jun;13(6):555-62. PubMed PMID: 16410826.

97. Nemunaitis J, Nemunaitis M, Senzer N, Snitz P, Bedell C, Kumar P, Pappen B, Maples PB, Shawler D, Fakhrai H. Phase II trial of Belagenpumatucel-L, a TGF-beta2 antisense gene modified allogeneic tumor vaccine in advanced non small cell lung cancer (NSCLC) patients. Cancer Gene Ther. 2009 Aug;16(8):620-4. doi: 10.1038/cgt.2009.15. Epub 2009 Mar 13. PubMed PMID: 19287371.

98. Nemunaitis J, Sterman D, Jablons D, Smith JW 2nd, Fox B, Maples P, Hamilton S, Borellini F, Lin A, Morali S, Hege K. Granulocyte-macrophage colony-stimulating factor gene-modified autologous tumor vaccines in non-small-cell lung cancer. J Natl Cancer Inst. 2004 Feb 18;96(4):326-31. PubMed PMID: 14970281.

99. Newton DA, Romano C, Gattoni-Celli S. Semiallogeneic cell hybrids as therapeutic vaccines for cancer. J Immunother. 2000 Mar-Apr;23(2):246-54. PubMed PMID: 10746551.

100. Ocadlíková D, Zahradová L, Kovárová L, Smejkalová J, Pour L, Vidláková P, Kyjovská D, Moravcová J, Rycová M, Novotná H, Jelínková I, Penka M, Michálek J, Hájek R. [The preparation of anticancer vaccine for patients with multiple myeloma on the base of monoclonal immunoglobulin loaded dendritic cells]. Klin Onkol. 2009;22(2):67-72. Czech. PubMed PMID: 19522376.

101. Ogasawara M, Miyashita M, Ota S. Vaccination of Urological Cancer Patients With WT1 Peptide-Pulsed Dendritic Cells in Combination With Molecular Targeted Therapy or Conventional Chemotherapy Induces Immunological and Clinical Responses. Ther Apher Dial. 2018 Jun;22(3):266-277.

102. Ogasawara M, Miyashita M, Yamagishi Y, Ota S. Phase I/II Pilot Study of Wilms' Tumor 1 Peptide-Pulsed Dendritic Cell Vaccination Combined With Conventional Chemotherapy in Patients With Head and Neck Cancer. Ther Apher Dial. 2019 Jun;23(3):279-288.
103. O'Rourke MG, Johnson M, Lanagan C, See J, Yang J, Bell JR, Slater GJ, Kerr BM, Crowe B, Purdie DM, Elliott SL, Ellem KA, Schmidt CW. Durable complete clinical responses in a phase I/II trial using an autologous melanoma cell/dendritic cell vaccine. Cancer Immunol Immunother. 2003 Jun;52(6):387-95. Epub 2003 Apr 8. PubMed PMID: 12682787.
104. Oshita C, Takikawa M, Kume A, Miyata H, Ashizawa T, Iizuka A, Kiyohara Y, Yoshikawa S, Tanosaki R, Yamazaki N, Yamamoto A, Takesako K, Yamaguchi K, Akiyama Y. Dendritic cell-based vaccination in metastatic melanoma patients: phase II clinical trial. Oncol Rep. 2012 Oct;28(4):1131-8. doi: 10.3892/or.2012.1956. Epub 2012 Aug 7. PubMed PMID: 22895835; PubMed Central PMCID: PMC3583475.
105. Pandha HS, John RJ, Hutchinson J, James N, Whelan M, Corbishley C, Dalgleish AG. Dendritic cell immunotherapy for urological cancers using cryopreserved allogeneic tumour lysate-pulsed cells: a phase I/II study. BJU Int. 2004 Aug;94(3):412-8. PubMed PMID: 15291878.
106. Pecher G, Häring A, Kaiser L, Thiel E. Mucin gene (MUC1) transfected dendritic cells as vaccine: results of a phase I/II clinical trial. Cancer Immunol Immunother. 2002 Dec;51(11-12):669-73. Epub 2002 Oct 19. PubMed PMID: 12439613.
107. Peng B, Liang L, Chen Z, He Q, Kuang M, Zhou F, Lu M, Huang J. Autologous tumor vaccine lowering postsurgical recurrent rate of hepatocellular carcinoma. Hepatogastroenterology. 2006 May-Jun;53(69):409-14. PubMed PMID: 16795983.
108. Peng BG, Liu SQ, Kuang M, He Q, Totsuka S, Huang L, Huang J, Lu MD, Liang LJ, Leong KW, Ohno T. Autologous fixed tumor vaccine: a formulation with cytokine-microparticles for protective immunity against recurrence of human hepatocellular carcinoma. Jpn J Cancer Res. 2002 Apr;93(4):363-8. PubMed PMID: 11985784; PubMed Central PMCID: PMC5927015.
109. Rains N, Cannan RJ, Chen W, Stubbs RS. Development of a dendritic cell (DC)-based vaccine for patients with advanced colorectal cancer. Hepatogastroenterology. 2001 Mar-Apr;48(38):347-51. PubMed PMID: 11379307.
110. Ribas A, Camacho LH, Lee SM, Hersh EM, Brown CK, Richards JM, Rodriguez MJ, Prieto VG, Glaspy JA, Oseguera DK, Hernandez J, Villanueva A, Chmielowski B, Mitsky P, Bercovici N, Wasserman E, Landais D, Ross MI. Multicenter phase II study of matured dendritic cells pulsed with melanoma cell line lysates in patients with advanced melanoma. J Transl Med. 2010 Sep 27;8:89. doi: 10.1186/1479-5876-8-89. PubMed PMID: 20875102; PubMed Central PMCID: PMC2954849.
111. Ribas A, Glaspy JA, Lee Y, Dissette VB, Seja E, Vu HT, Tchekmedyian NS, Oseguera D, Comin-Anduix B, Wargo JA, Amarnani SN, McBride WH, Economou JS, Butterfield LH. Role of dendritic cell phenotype, determinant spreading, and negative costimulatory blockade in dendritic cell-based melanoma immunotherapy. J Immunother. 2004 Sep-Oct;27(5):354-67. PubMed PMID: 15314544.

112. Ridolfi L, Petrini M, Fiammenghi L, Granato AM, Ancarani V, Pancisi E, Scarpi E, Guidoboni M, Migliori G, Sanna S, Tauceri F, Verdecchia GM, Riccobon A, Valmorri L, Ridolfi R. Unexpected high response rate to traditional therapy after dendritic cell-based vaccine in advanced melanoma: update of clinical outcome and subgroup analysis. Clin Dev Immunol. 2010;2010:504979. doi: 10.1155/2010/504979. Epub 2010 Sep 27. Erratum in: Clin Dev Immunol. 2011;2011:283896. Valmorri, Linda [added]. PubMed PMID: 20936106; PubMed Central PMCID: PMC2948909.
113. Rini BI, Weinberg V, Fong L, Conry S, Hershberg RM, Small EJ. Combination immunotherapy with prostatic acid phosphatase pulsed antigen-presenting cells (provenge) plus bevacizumab in patients with serologic progression of prostate cancer after definitive local therapy. Cancer. 2006 Jul 1;107(1):67-74. PubMed PMID: 16736512.
114. Roddie H, Klammer M, Thomas C, Thomson R, Atkinson A, Sproul A, Waterfall M, Samuel K, Yin J, Johnson P, Turner M. Phase I/II study of vaccination with dendritic-like leukaemia cells for the immunotherapy of acute myeloid leukaemia. Br J Haematol. 2006 Apr;133(2):152-7. PubMed PMID: 16611305.
115. Rodriguez J, Castañón E, Perez-Gracia JL, Rodriguez I, Viudez A, Alfaro C, Oñate C, Perez G, Rotellar F, Inogés S, López-Diaz de Cerio A, Resano L, Ponz-Sarvise M, Rodriguez-Ruiz ME, Chopitea A, Vera R, Melero I. A randomized phase II clinical trial of dendritic cell vaccination following complete resection of colon cancer liver metastasis. J Immunother Cancer. 2018 Sep 29;6(1):96.
116. Sakakibara M, Kanto T, Hayakawa M, Kuroda S, Miyatake H, Itose I, Miyazaki M, Kakita N, Higashitani K, Matsubara T, Hiramatsu N, Kasahara A, Takehara T, Hayashi N. Comprehensive immunological analyses of colorectal cancer patients in the phase I/II study of quickly matured dendritic cell vaccine pulsed with carcinoembryonic antigen peptide. Cancer Immunol Immunother. 2011 Nov;60(11):1565-75. doi: 10.1007/s00262-011-1051-1. Epub 2011 Jun 17. PubMed PMID: 21681375.
117. Salcedo M, Bercovici N, Taylor R, Vereecken P, Massicard S, Duriau D, Vernel-Pauillac F, Boyer A, Baron-Bodo V, Mallard E, Bartholeyns J, Goxe B, Latour N, Leroy S, Prigent D, Martiat P, Sales F, Laporte M, Bruyns C, Romet-Lemonne JL, Abastado JP, Lehmann F, Velu T. Vaccination of melanoma patients using dendritic cells loaded with an allogeneic tumor cell lysate. Cancer Immunol Immunother. 2006 Jul;55(7):819-29. Epub 2005 Sep 27. PubMed PMID: 16187085.
118. Santegoets SJ, Stam AG, Lougheed SM, Gall H, Scholten PE, Reijm M, Jooss K, Sacks N, Hege K, Lowy I, Cuillerot JM, von Blomberg BM, Scheper RJ, van den Eertwegh AJ, Gerritsen WR, de Gruijl TD. T cell profiling reveals high CD4+CTLA-4 + T cell frequency as dominant predictor for survival after prostate GVAX/ipilimumab treatment. Cancer Immunol Immunother. 2013 Feb;62(2):245-56. doi: 10.1007/s00262-012-1330-5. Epub 2012 Aug 10. PubMed PMID: 22878899.
119. Schlag P, Manasterski M, Gerneth T, Hohenberger P, Dueck M, Herfarth C, Liebrich W, Schirrmacher V. Active specific immunotherapy with Newcastle-disease-virus-modified autologous tumor cells following resection of liver metastases in colorectal cancer. First evaluation of clinical response of a phase II-trial. Cancer Immunol Immunother. 1992;35(5):325-30. PubMed PMID: 1394336.

120. Schreibelt G, Bol KF, Westdorp H, Wimmers F, Aarntzen EH, Duiveman-de Boer T, van de Rakt MW, Scharenborg NM, de Boer AJ, Pots JM, Olde Nordkamp MA, van Oorschot TG, Tel J, Winkels G, Petry K, Blokx WA, van Rossum MM, Welzen ME, Mus RD, Croockewit SA, Koornstra RH, Jacobs JF, Kelderman S, Blank CU, Gerritsen WR, Punt CJ, Figdor CG, de Vries IJ. Effective Clinical Responses in Metastatic Melanoma Patients after Vaccination with Primary Myeloid Dendritic Cells. Clin Cancer Res. 2016 May 1;22(9):2155-66. doi: 10.1158/1078-0432.CCR-15-2205. Epub 2015 Dec 28. PubMed PMID: 26712687.
121. Schwaab T, Heaney JA, Schned AR, Harris RD, Cole BF, Noelle RJ, Phillips DM, Stempkowski L, Ernstoff MS. A randomized phase II trial comparing two different sequence combinations of autologous vaccine and human recombinant interferon gamma and human recombinant interferon alpha2B therapy in patients with metastatic renal cell carcinoma: clinical outcome and analysis of immunological parameters. J Urol. 2000 Apr;163(4):1322-7. PubMed PMID: 10737537.
122. Schwaab T, Schwarzer A, Wolf B, Crocenzi TS, Seigne JD, Crosby NA, Cole BF, Fisher JL, Uhlenhake JC, Mellinger D, Foster C, Szczepiorkowski ZM, Webber SM, Schned AR, Harris RD, Barth RJ Jr, Heaney JA, Noelle RJ, Ernstoff MS. Clinical and immunologic effects of intranodal autologous tumor lysate-dendritic cell vaccine with Aldesleukin (Interleukin 2) and IFN-{alpha}2a therapy in metastatic renal cell carcinoma patients. Clin Cancer Res. 2009 Aug 1;15(15):4986-92. doi: 10.1158/1078-0432.CCR-08-3240. Epub 2009 Jul 21. PubMed PMID: 19622576; PubMed Central PMCID: PMC3775650.
123. Simmons SJ, Tjoa BA, Rogers M, Elgamal A, Kenny GM, Ragde H, Troychak MJ, Boynton AL, Murphy GP. GM-CSF as a systemic adjuvant in a phase II prostate cancer vaccine trial. Prostate. 1999 Jun 1;39(4):291-7. PubMed PMID: 10344219.
124. Simons JW, Carducci MA, Mikhak B, Lim M, Biedrzycki B, Borellini F, Clift SM, Hege KM, Ando DG, Piantadosi S, Mulligan R, Nelson WG. Phase I/II trial of an allogeneic cellular immunotherapy in hormone-naïve prostate cancer. Clin Cancer Res. 2006 Jun 1;12(11 Pt 1):3394-401. PubMed PMID: 16740763.
125. Small EJ, Fratesi P, Reese DM, Strang G, Laus R, Peshwa MV, Valone FH. Immunotherapy of hormone-refractory prostate cancer with antigen-loaded dendritic cells. J Clin Oncol. 2000 Dec 1;18(23):3894-903. PubMed PMID: 11099318.
126. Smithers M, O'Connell K, MacFadyen S, Chambers M, Greenwood K, Boyce A, Abdul-Jabbar I, Barker K, Grimmett K, Walpole E, Thomas R. Clinical response after intradermal immature dendritic cell vaccination in metastatic melanoma is associated with immune response to particulate antigen. Cancer Immunol Immunother. 2003 Jan;52(1):41-52. Epub 2002 Nov 13. PubMed PMID: 12536239.
127. Soleimani A, Berntsen A, Svane IM, Pedersen AE. Immune responses in patients with metastatic renal cell carcinoma treated with dendritic cells pulsed with tumor lysate. Scand J Immunol. 2009 Nov;70(5):481-9. doi: 10.1111/j.1365-3083.2009.02322.x. PubMed PMID: 19874553.
128. Spaner DE, Hammond C, Mena J, Foden C, Deabreu A. A phase I/II trial of oxidized autologous tumor vaccines during the "watch and wait" phase of chronic lymphocytic

leukemia. Cancer Immunol Immunother. 2005 Jul;54(7):635-46. Epub 2004 Dec 17. PubMed PMID: 15918075.

129. Steele JC, Rao A, Marsden JR, Armstrong CJ, Berhane S, Billingham LJ, Graham N, Roberts C, Ryan G, Uppal H, Walker C, Young LS, Steven NM. Phase I/II trial of a dendritic cell vaccine transfected with DNA encoding melan A and gp100 for patients with metastatic melanoma. Gene Ther. 2011 Jun;18(6):584-93. doi: 10.1038/gt.2011.1. Epub 2011 Feb 10. PubMed PMID: 21307889.

130. Svane IM, Pedersen AE, Nikolajsen K, Zocca MB. Alterations in p53-specific T cells and other lymphocyte subsets in breast cancer patients during vaccination with p53-peptide loaded dendritic cells and low-dose interleukin-2. Vaccine. 2008 Aug 26;26(36):4716-24. doi: 10.1016/j.vaccine.2008.06.085. Epub 2008 Jul 9. PubMed PMID: 18616968.

131. Tada F, Abe M, Hirooka M, Ikeda Y, Hiasa Y, Lee Y, Jung NC, Lee WB, Lee HS, Bae YS, Onji M. Phase I/II study of immunotherapy using tumor antigen-pulsed dendritic cells in patients with hepatocellular carcinoma. Int J Oncol. 2012 Nov;41(5):1601-9. doi: 10.3892/ijo.2012.1626. Epub 2012 Sep 11. PubMed PMID: 22971679; PubMed Central PMCID: PMC3583872.

132. Thomas-Kaskel AK, Zeiser R, Jochim R, Robbel C, Schultze-Seemann W, Waller CF, Veelken H. Vaccination of advanced prostate cancer patients with PSCA and PSA peptide-loaded dendritic cells induces DTH responses that correlate with superior overall survival. Int J Cancer. 2006 Nov 15;119(10):2428-34. PubMed PMID: 16977630.

133. Tjoa BA, Lodge PA, Salgaller ML, Boynton AL, Murphy GP. Dendritic cell-based immunotherapy for prostate cancer. CA Cancer J Clin. 1999 Mar-Apr;49(2):117-28, 65. PubMed PMID: 11198886.

134. Trefzer U, Weingart G, Chen Y, Herberth G, Adrian K, Winter H, Audring H, Guo Y, Sterry W, Walden P. Hybrid cell vaccination for cancer immune therapy: first clinical trial with metastatic melanoma. Int J Cancer. 2000 Mar 1;85(5):618-26. PubMed PMID: 10699939.

135. Trepiakas R, Berntsen A, Hadrup SR, Bjørn J, Geertsen PF, Straten PT, Andersen MH, Pedersen AE, Soleimani A, Lorentzen T, Johansen JS, Svane IM. Vaccination with autologous dendritic cells pulsed with multiple tumor antigens for treatment of patients with malignant melanoma: results from a phase I/II trial. Cytotherapy. 2010 Oct;12(6):721-34. doi: 10.3109/14653241003774045. PubMed PMID: 20429791.

136. Tsioulias GJ, Gupta RK, Tisman G, Hsueh EC, Essner R, Wanek LA, Morton DL. Serum TA90 antigen-antibody complex as a surrogate marker for the efficacy of a polyvalent allogeneic whole-cell vaccine (CancerVax) in melanoma. Ann Surg Oncol. 2001 Apr;8(3):198-203. PubMed PMID: 11314934.

137. Urba WJ, Nemunaitis J, Marshall F, Smith DC, Hege KM, Ma J, Nguyen M, Small EJ. Treatment of biochemical recurrence of prostate cancer with granulocyte-macrophage colony-stimulating factor secreting, allogeneic, cellular immunotherapy. J Urol. 2008 Nov;180(5):2011-7; discussion 2017-8. doi: 10.1016/j.juro.2008.07.048. Epub 2008 Sep 17. PubMed PMID: 18801509.

138. Vaishampayan U, Abrams J, Darrah D, Jones V, Mitchell MS. Active immunotherapy of metastatic melanoma with allogeneic melanoma lysates and interferon alpha. Clin Cancer Res. 2002 Dec;8(12):3696-701. Review. PubMed PMID: 12473578.
139. Van Tendeloo VF, Van de Velde A, Van Driessche A, Cools N, Anguille S, Ladell K, Gostick E, Vermeulen K, Pieters K, Nijs G, Stein B, Smits EL, Schroyens WA, Gadisseur AP, Vrelust I, Jorens PG, Goossens H, de Vries IJ, Price DA, Oji Y, Oka Y, Sugiyama H, Berneman ZN. Induction of complete and molecular remissions in acute myeloid leukemia by Wilms' tumor 1 antigen-targeted dendritic cell vaccination. Proc Natl Acad Sci U S A. 2010 Aug 3;107(31):13824-9. doi: 10.1073/pnas.1008051107. Epub 2010 Jul 14. PubMed PMID: 20631300; PubMed Central PMCID: PMC2922237.
140. Waeckerle-Men Y, Uetz-von Allmen E, Fopp M, von Moos R, Böhme C, Schmid HP, Ackermann D, Cerny T, Ludewig B, Groettrup M, Gillessen S. Dendritic cell-based multi-epitope immunotherapy of hormone-refractory prostate carcinoma. Cancer Immunol Immunother. 2006 Dec;55(12):1524-33. Epub 2006 Apr 13. PubMed PMID: 16612599.
141. Whiteside TL, Zhao Y, Tsukishiro T, Elder EM, Gooding W, Baar J. Enzyme-linked immunospot, cytokine flow cytometry, and tetramers in the detection of T-cell responses to a dendritic cell-based multipeptide vaccine in patients with melanoma. Clin Cancer Res. 2003 Feb;9(2):641-9. PubMed PMID: 12576430.
142. Wierecky J, Mueller M, Brossart P. Dendritic cell-based cancer immunotherapy targeting MUC-1. Cancer Immunol Immunother. 2006 Jan;55(1):63-7. Epub 2005 Oct 27. PubMed PMID: 15864588.
143. Wierecky J, Müller MR, Wirths S, Halder-Oehler E, Dörfel D, Schmidt SM, Häntschel M, Brugger W, Schröder S, Horger MS, Kanz L, Brossart P. Immunologic and clinical responses after vaccinations with peptide-pulsed dendritic cells in metastatic renal cancer patients. Cancer Res. 2006 Jun 1;66(11):5910-8. PubMed PMID: 16740731.
144. Wittig B, Märten A, Dorbic T, Weineck S, Min H, Niemitz S, Trojaneck B, Flieger D, Kruopis S, Albers A, Löffel J, Neubauer A, Albers P, Müller S, Sauerbruch T, Bieber T, Huhn D, Schmidt-Wolf IG. Therapeutic vaccination against metastatic carcinoma by expression-modulated and immunomodified autologous tumor cells: a first clinical phase I/II trial. Hum Gene Ther. 2001 Feb 10;12(3):267-78. PubMed PMID: 11177563.
145. Yamanaka R, Abe T, Yajima N, Tsuchiya N, Homma J, Kobayashi T, Narita M, Takahashi M, Tanaka R. Vaccination of recurrent glioma patients with tumour lysate-pulsed dendritic cells elicits immune responses: results of a clinical phase I/II trial. Br J Cancer. 2003 Oct 6;89(7):1172-9. PubMed PMID: 14520441; PubMed Central PMCID: PMC2394324.
146. Yamanaka R, Homma J, Yajima N, Tsuchiya N, Sano M, Kobayashi T, Yoshida S, Abe T, Narita M, Takahashi M, Tanaka R. Clinical evaluation of dendritic cell vaccination for patients with recurrent glioma: results of a clinical phase I/II trial. Clin Cancer Res. 2005 Jun 1;11(11):4160-7. PubMed PMID: 15930352.
147. Yao Y, Luo F, Tang C, Chen D, Qin Z, Hua W, Xu M, Zhong P, Yu S, Chen D, Ding X, Zhang Y, Zheng X, Yang J, Qian J, Deng Y, Hoon DSB, Hu J, Chu Y, Zhou L. Molecular subgroups and B7-H4 expression levels predict responses to dendritic cell vaccines in glioblastoma: an exploratory randomized phase II clinical trial. Cancer Immunol Immunother. 2018 Nov;67(11):1777-1788.

148. Zhang W, Lu X, Cui P, Piao C, Xiao M, Liu X, Wang Y, Wu X, Liu J, Yang L. Phase I/II clinical trial of a Wilms' tumor 1-targeted dendritic cell vaccination-based immunotherapy in patients with advanced cancer. Cancer Immunol Immunother. 2019 Jan;68(1):121-130.

Phase III Studies

1. Beer TM, Bernstein GT, Corman JM, et al. Randomized trial of autologous cellular immunotherapy with sipuleucel-T in androgen-dependent prostate cancer. Clin Cancer Res. 2011;17: 4558-4567
2. Carson WE 3rd, Unger JM, Sosman JA, Flaherty LE, Tuthill RJ, Porter MJ, Thompson JA, Kempf RA, Othus M, Ribas A, Sondak VK. Adjuvant vaccine immunotherapy of resected, clinically node-negative melanoma: long-term outcome and impact of HLA class I antigen expression on overall survival. Cancer Immunol Res. 2014 Oct;2(10):981-7. doi: 10.1158/2326-6066.CIR-14-0052. Epub 2014 Jul 3. PubMed PMID: 24994597; PubMed Central PMCID: PMC4185232.
3. Doehn Ch, Richter A, Lehmacher W, Jocham D. Adjuvant autologous tumour cell-lysate vaccine versus no adjuvant treatment in patients with M0 renal cell carcinoma after radical nephrectomy: 3-year interim analysis of a German multicentre phase-III trial. Folia Biol (Praha). 2003;49(2):69-73. PubMed PMID: 12779015.
4. George DJ, Nabhan C, DeVries T, Whitmore JB, Gomella LG. Survival Outcomes of Sipuleucel-T Phase III Studies: Impact of Control-Arm Cross-Over to Salvage Immunotherapy. Cancer Immunol Res. 2015 Sep;3(9):1063-9. doi: 10.1158/2326-6066.CIR-15-0006. Epub 2015 May 5. PubMed PMID: 25943532.
5. Giaccone G, Bazhenova LA, Nemunaitis J, Tan M, Juhász E, Ramlau R, van den Heuvel MM, Lal R, Kloecker GH, Eaton KD, Chu Q, Dunlop DJ, Jain M, Garon EB, Davis CS, Carrier E, Moses SC, Shawler DL, Fakhrai H. A phase III study of belagenpumatucel-L, an allogeneic tumour cell vaccine, as maintenance therapy for non-small cell lung cancer. Eur J Cancer. 2015 Nov;51(16):2321-9. doi: 10.1016/j.ejca.2015.07.035. Epub 2015 Aug 14. PubMed PMID: 26283035.
6. Hanna MG Jr, Hoover HC Jr, Vermorken JB, Harris JE, Pinedo HM. Adjuvant active specific immunotherapy of stage II and stage III colon cancer with an autologous tumor cell vaccine: first randomized phase III trials show promise. Vaccine. 2001 Mar 21;19(17-19):2576-82. PubMed PMID: 11257395.
7. Higano CS, Schellhammer PF, Small EJ, Burch PA, Nemunaitis J, Yuh L, Provost N, Frohlich MW. Integrated data from 2 randomized, double-blind, placebo-controlled, phase 3 trials of active cellular immunotherapy with sipuleucel-T in advanced prostate cancer. Cancer. 2009 Aug 15;115(16):3670-9. doi: 10.1002/cncr.24429. PubMed PMID: 19536890.
8. Hoover HC Jr, Brandhorst JS, Peters LC, Surdyke MG, Takeshita Y, Madariaga J, Muenz LR, Hanna MG Jr. Adjuvant active specific immunotherapy for human colorectal cancer:

6.5-year median follow-up of a phase III prospectively randomized trial. J Clin Oncol. 1993 Mar;11(3):390-9. PubMed PMID: 8445413.
9. Jocham D, Richter A, Hoffmann L, Iwig K, Fahlenkamp D, Zakrzewski G, Schmitt E, Dannenberg T, Lehmacher W, von Wietersheim J, Doehn C. Adjuvant autologous renal tumour cell vaccine and risk of tumour progression in patients with renal-cell carcinoma after radical nephrectomy: phase III, randomised controlled trial. Lancet. 2004 Feb 21;363(9409):594-9. PubMed PMID: 14987883.
10. Kantoff PW, Higano CS, Shore ND, Berger ER, Small EJ, Penson DF, Redfern CH, Ferrari AC, Dreicer R, Sims RB, Xu Y, Frohlich MW, Schellhammer PF; IMPACT Study Investigators. Sipuleucel-T immunotherapy for castration-resistant prostate cancer. N Engl J Med. 2010 Jul 29;363(5):411-22. doi: 10.1056/NEJMoa1001294. PubMed PMID: 20818862.
11. Liau LM, Ashkan K, Tran DD, Campian JL, Trusheim JE, Cobbs CS, Heth JA, Salacz M, Taylor S, D'Andre SD, Iwamoto FM, Dropcho EJ, Moshel YA, Walter KA, Pillainayagam CP, Aiken R, Chaudhary R, Goldlust SA, Bota DA, Duic P, Grewal J, Elinzano H, Toms SA, Lillehei KO, Mikkelsen T, Walbert T, Abram SR, Brenner AJ, Brem S, Ewend MG, Khagi S, Portnow J, Kim LJ, Loudon WG, Thompson RC, Avigan DE, Fink KL, Geoffroy FJ, Lindhorst S, Lutzky J, Sloan AE, Schackert G, Krex D, Meisel HJ, Wu J, Davis RP, Duma C, Etame AB, Mathieu D, Kesari S, Piccioni D, Westphal M, Baskin DS, New PZ, Lacroix M, May SA, Pluard TJ, Tse V, Green RM, Villano JL, Pearlman M, Petrecca K, Schulder M, Taylor LP, Maida AE, Prins RM, Cloughesy TF, Mulholland P, Bosch ML. First results on survival from a large Phase 3 clinical trial of an autologous dendritic cell vaccine in newly diagnosed glioblastoma. J Transl Med. 2018 May 29;16(1):142. doi: 10.1186/s12967-018-1507-6. Erratum in: J Transl Med. 2018 Jun 29;16(1):179. PubMed PMID: 29843811; PubMed Central PMCID: PMC5975654.
12. Schadendorf D, Ugurel S, Schuler-Thurner B, Nestle FO, Enk A, Bröcker EB, Grabbe S, Rittgen W, Edler L, Sucker A, Zimpfer-Rechner C, Berger T, Kamarashev J, Burg G, Jonuleit H, Tüttenberg A, Becker JC, Keikavoussi P, Kämpgen E, Schuler G; DC study group of the DeCOG. Dacarbazine (DTIC) versus vaccination with autologous peptide-pulsed dendritic cells (DC) in first-line treatment of patients with metastatic melanoma: a randomized phase III trial of the DC study group of the DeCOG. Ann Oncol. 2006 Apr;17(4):563-70. Epub 2006 Jan 17. PubMed PMID: 16418308.
13. Skachkova OV, Khranovska NM, Gorbach OI, Svergun NM, Sydor RI, Nikulina VV. Immunological markers of anti-tumor dendritic cells vaccine efficiency in patients with non-small cell lung cancer. Exp Oncol. 2013 Jun;35(2):109-13. PubMed PMID: 23828386.
14. Sondak VK, Liu PY, Tuthill RJ, Kempf RA, Unger JM, Sosman JA, Thompson JA, Weiss GR, Redman BG, Jakowatz JG, Noyes RD, Flaherty LE. Adjuvant immunotherapy of resected, intermediate-thickness, node-negative melanoma with an allogeneic tumor vaccine: overall results of a randomized trial of the Southwest Oncology Group. J Clin Oncol. 2002 Apr 15;20(8):2058-66. PubMed PMID: 11956266.

15. Uyl-de Groot CA, Vermorken JB, Hanna MG Jr, Verboom P, Groot MT, Bonsel GJ, Meijer CJ, Pinedo HM. Immunotherapy with autologous tumor cell-BCG vaccine in patients with colon cancer: a prospective study of medical and economic benefits. Vaccine. 2005 Mar 18;23(17-18):2379-87. PubMed PMID: 15755632.
16. Wallack MK, Sivanandham M, Balch CM, Urist MM, Bland KI, Murray D, Robinson WA, Flaherty L, Richards JM, Bartolucci AA, Rosen L. Surgical adjuvant active specific immunotherapy for patients with stage III melanoma: the final analysis of data from a phase III, randomized, double-blind, multicenter vaccinia melanoma oncolysate trial. J Am Coll Surg. 1998 Jul;187(1):69-77; discussion 77-9. PubMed PMID: 9660028.
17. Wallack MK, Sivanandham M, Balch CM, Urist MM, Bland KI, Murray D, Robinson WA, Flaherty LE, Richards JM, Bartolucci AA, et al. A phase III randomized, double-blind multiinstitutional trial of vaccinia melanoma oncolysate-active specific immunotherapy for patients with stage II melanoma. Cancer. 1995 Jan 1;75(1):34-42. PubMed PMID: 7804974.
18. Wood C, Srivastava P, Bukowski R, Lacombe L, Gorelov AI, Gorelov S, Mulders P, Zielinski H, Hoos A, Teofilovici F, Isakov L, Flanigan R, Figlin R, Gupta R, Escudier B; C-100-12 RCC Study Group. An adjuvant autologous therapeutic vaccine (HSPPC-96; vitespen) versus observation alone for patients at high risk of recurrence after nephrectomy for renal cell carcinoma: a multicentre, open-label, randomised phase III trial. Lancet. 2008 Jul 12;372(9633):145-54. doi: 10.1016/S0140-6736(08)60697-2. Epub 2008 Jul 3. PubMed PMID: 18602688.
