# Supplementary material for: Regression of Breast Cancer Metastases Following Treatment with Irradiated SV-BR-1-GM, a GM-CSF Overexpressing Breast Cancer Cell Line: Intellectual Property and Immune Markers of Response
Source: Recent Pat Anticancer Drug Discov. 2023 Dec 28;18(2):224–40. doi: 10.2174/1574892817666220518123331 (PMC10009895; doi:10.2174/1574892817666220518123331)
Supplement: Supplementary file 1 [file PRA-18-224_SD1.zip › PRA-18-224_SD1/Wiseman_et_al_DataSheet_4_Final_PRA_post_review.pdf]

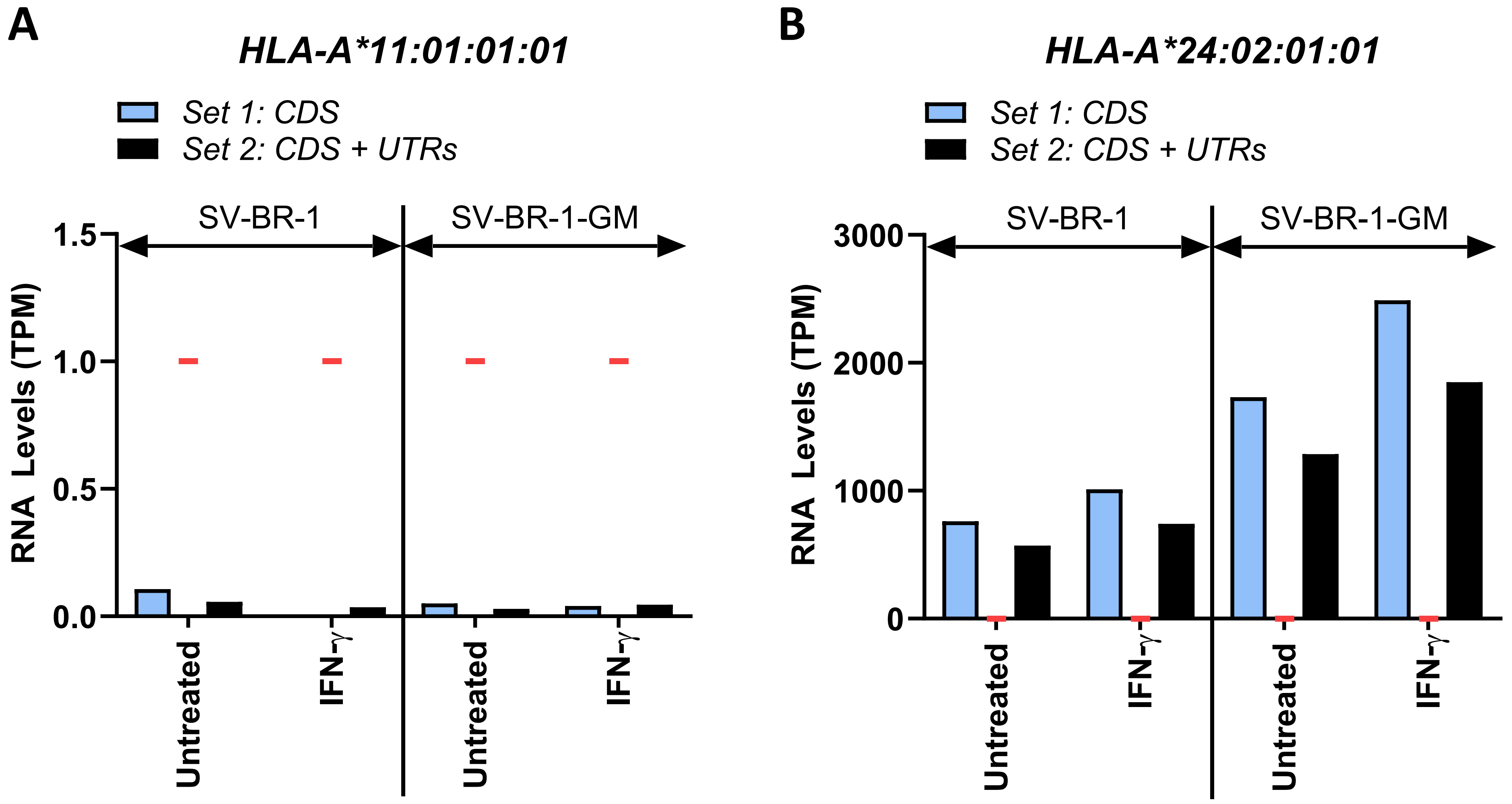
A
HLA-A*11:01:01:01
Set 1: CDS
Set 2: CDS + UTRs
SV-BR-1
SV-BR-1-GM
RNA Levels (TPM)
1.5
1.0
0.5
0.0
Untreated
IFN-γ
Untreated
IFN-γ
B
HLA-A*24:02:01:01
Set 1: CDS
Set 2: CDS + UTRs
SV-BR-1
SV-BR-1-GM
RNA Levels (TPM)
3000
2000
1000
0
Untreated
IFN-γ
Untreated
IFN-γ

Figure S1

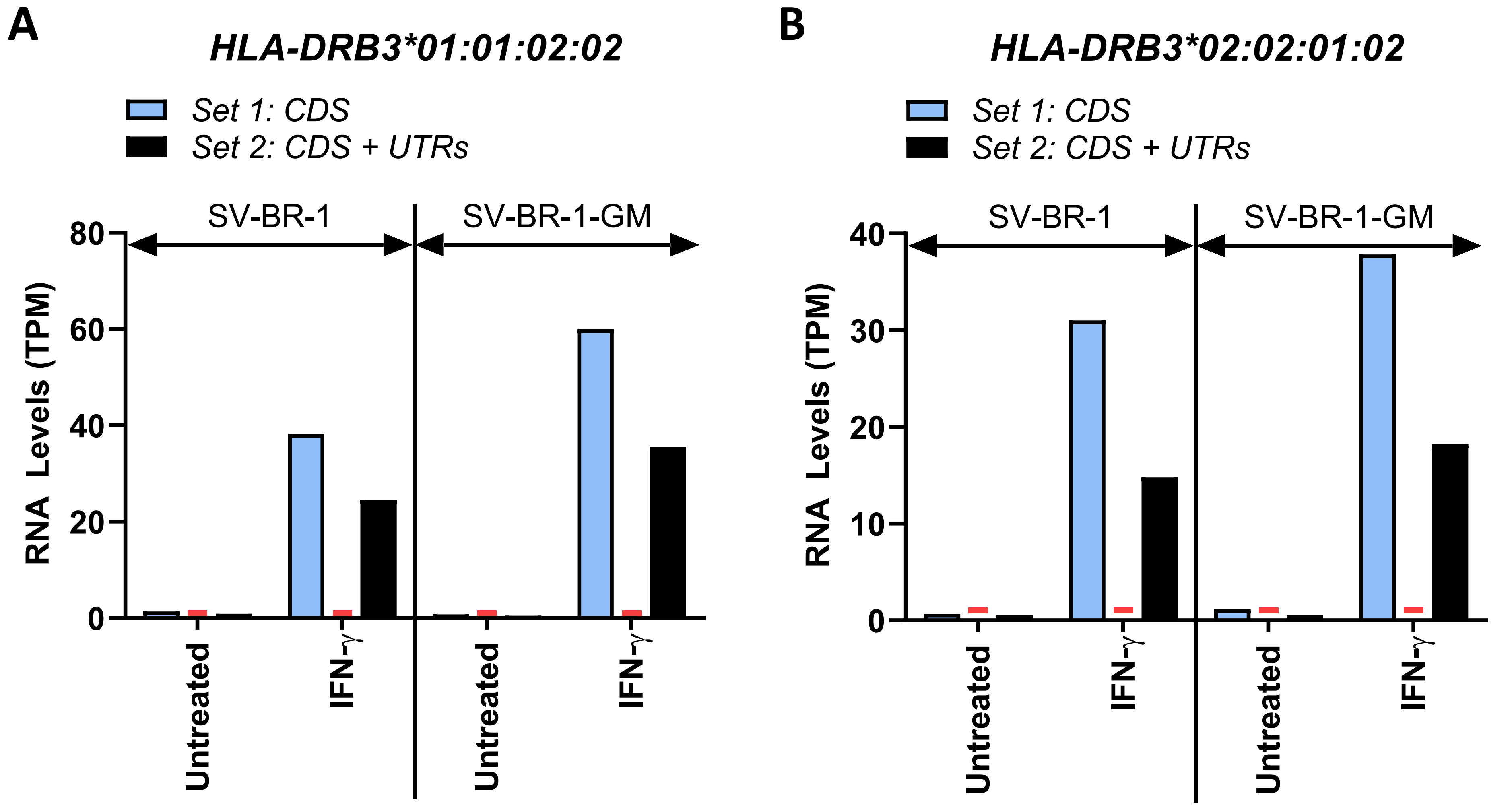

A
HLA-DRB3*01:01:02:02
Set 1: CDS
Set 2: CDS + UTRs
SV-BR-1
SV-BR-1-GM
RNA Levels (TPM)
80
60
40
20
0
Untreated
IFN-γ
Untreated
IFN-γ
B
HLA-DRB3*02:02:01:02
Set 1: CDS
Set 2: CDS + UTRs
SV-BR-1
SV-BR-1-GM
RNA Levels (TPM)
40
30
20
10
0
Untreated
IFN-γ
Untreated
IFN-γ

Figure S2

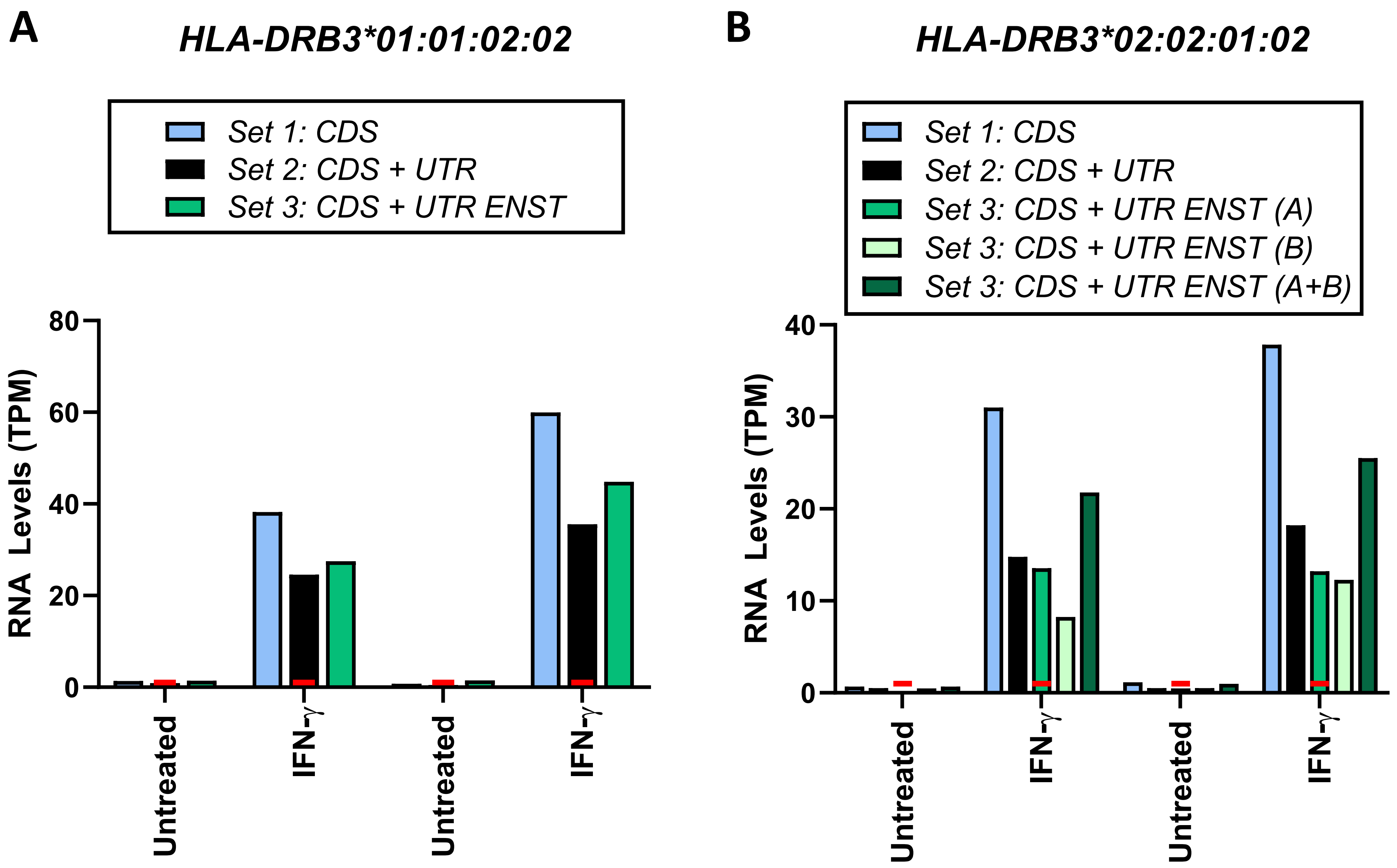
A
HLA-DRB3*01:01:02:02
Set 1: CDS
Set 2: CDS + UTR
Set 3: CDS + UTR ENST
RNA Levels (TPM)
80
60
40
20
0
Untreated
IFN-γ
Untreated
IFN-γ
B
HLA-DRB3*02:02:01:02
Set 1: CDS
Set 2: CDS + UTR
Set 3: CDS + UTR ENST (A)
Set 3: CDS + UTR ENST (B)
Set 3: CDS + UTR ENST (A+B)
RNA Levels (TPM)
40
30
20
10
0
Untreated
IFN-γ
Untreated
IFN-γ

Figure S3

Gel electrophoresis to validate qPCR Primer Specificity

*HLA-DRB3*01:01* primer set (after 40 cycles)

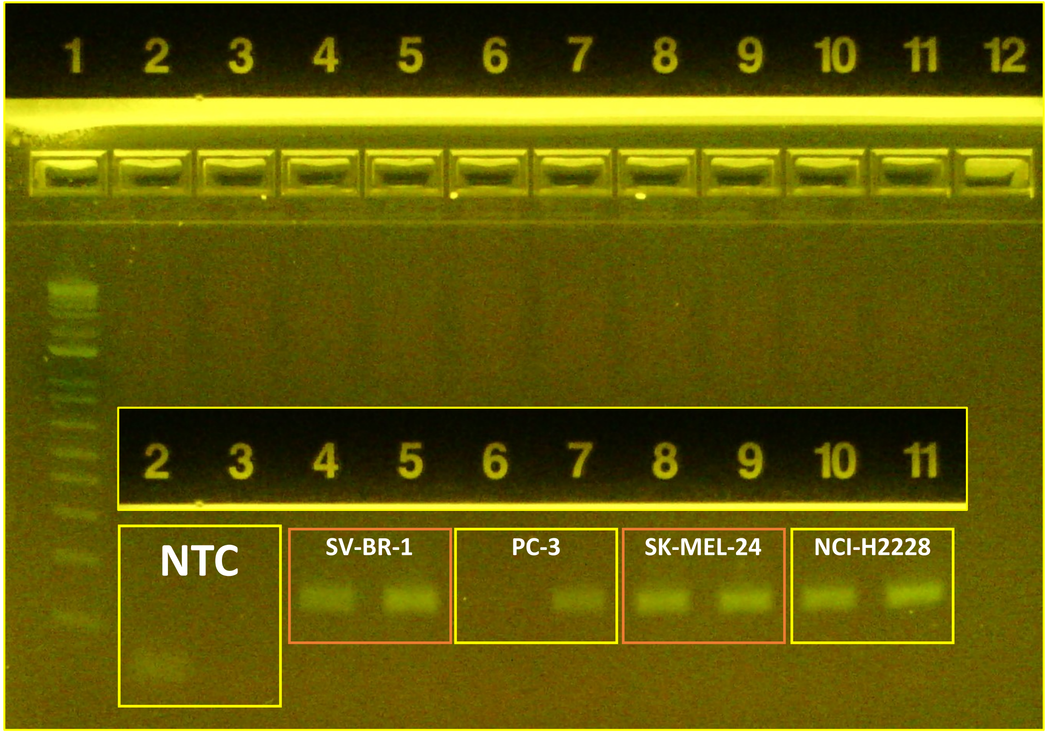

4, 6, 8, 10 – Vehicle treated
5, 7 ,9, 11 – IFN-γ treated
NTC: no template control (no cDNA)

Figure S4

Tumor regression in Breast in Subject A002

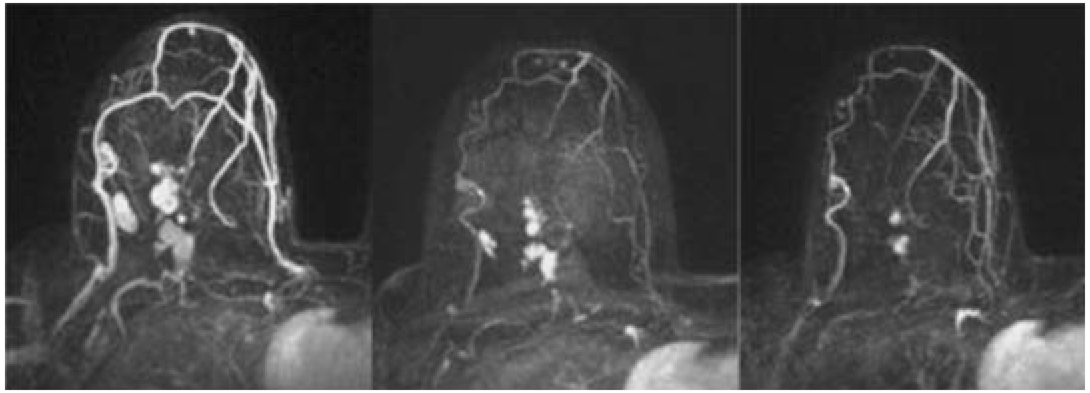

Published in: Wiseman, C.L., and Kharazi, A. (2006). Objective clinical regression of metastatic breast cancer in disparate sites after use of whole-cell vaccine genetically modified to release sargramostim. *Breast J* 12**,** 475-480. doi: 10.1111/j.1075-122X.2006.00319.x

Used with kind permission from John Wiley and Sons, Inc.

Figure S5
